# Sex-Specific Transcriptomic Changes in the Villous Tissue of Placentas of Pregnant Women Using a Selective Serotonin Reuptake Inhibitor

**DOI:** 10.1021/acschemneuro.3c00621

**Published:** 2024-02-29

**Authors:** Laura Staal, Torsten Plösch, Theodora Kunovac Kallak, Inger Sundström Poromaa, Bregje Wertheim, Jocelien D. A. Olivier

**Affiliations:** † Neurobiology, Groningen Institute for Evolutionary Life Sciences, University of Groningen, 9700 CC Groningen, The Netherlands; ‡ Department of Cardiology, University Medical Center Groningen, University of Groningen, 9713 GZ Groningen, The Netherlands; § Departments of Obstetrics and Gynaecology, University Medical Center Groningen, University of Groningen, 9713 GZ Groningen, The Netherlands; ∥ Perinatal Neurobiology, Department of Human Medicine, School of Medicine and Health Sciences, Carl von Ossietzky University Oldenburg, 26129 Oldenburg, Germany; ⊥ Evolutionary Genetics, Development & Behaviour, Groningen Institute for Evolutionary Life Sciences, University of Groningen, 9700 CC Groningen, The Netherlands; # Department of Women’s and Children’s Health, Uppsala University, 75185 Uppsala, Sweden

**Keywords:** SSRI, antidepressant, pregnancy, placenta, sex difference

## Abstract

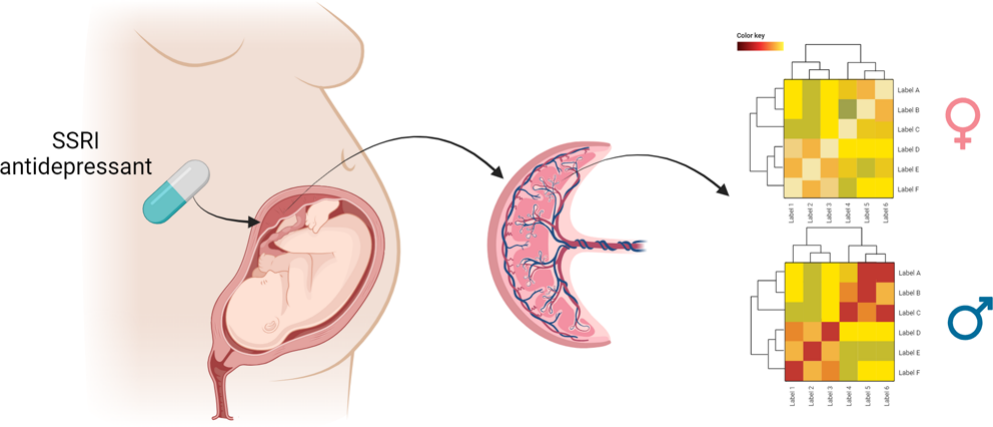

About 5% of pregnant women are treated with selective serotonin reuptake inhibitor (SSRI) antidepressants to treat their depression. SSRIs influence serotonin levels, a key factor in neural embryonic development, and their use during pregnancy has been associated with adverse effects on the developing embryo. However, the role of the placenta in transmitting these negative effects is not well understood. In this study, we aim to elucidate how disturbances in the maternal serotonergic system affect the villous tissue of the placenta by assessing whole transcriptomes in the placentas of women with healthy pregnancies and women with depression and treated with the SSRI fluoxetine during pregnancy. Twelve placentas of the Biology, Affect, Stress, Imaging and Cognition in Pregnancy and the Puerperium (BASIC) project were selected for RNA sequencing to examine differentially expressed genes: six male infants and six female infants, equally distributed over women treated with SSRI and without SSRI treatment. Our results show that more genes in the placenta of male infants show changed expression associated with fluoxetine treatment than in placentas of female infants, stressing the importance of sex-specific analyses. In addition, we identified genes related to extracellular matrix organization to be significantly enriched in placentas of male infants born to women treated with fluoxetine. It remains to be established whether the differentially expressed genes that we found to be associated with SSRI treatment are the result of the SSRI treatment itself, the underlying depression, or a combination of the two.

## Introduction

Pregnancy is often portrayed as a time of great joy, but unfortunately that is not the reality for all women. Depressive symptoms during pregnancy are not uncommon. In fact, 20% of women experience some symptoms of depression at any time of their pregnancy.^1^ Around 5% of pregnant women suffer from major depression and pharmacological treatment is often unavoidable.^2,3^ The most prescribed antidepressants are selective serotonin reuptake inhibitors (SSRIs) because of their high efficacy, few side effects, and therapeutic safety.^4^ The use of SSRIs increased significantly when fluoxetine (Prozac, the first SSRI) was released in the market (late 1980s), and quickly became the most widely prescribed drug in North America, and second worldwide.^5^ The use of SSRIs during pregnancy in both Europe and the U.S. has increased tremendously over the past decades.^6−9^ In Europe, 2.5–3.3% of pregnant women use SSRIs,^10,11^ while in the U.S. the occurrence is between 2.7 and 5.4%.^12,13^

SSRIs are considered safe for antenatal use as they do not cause gross teratogenic effects.^14^ However, studies report that the use of SSRIs during pregnancy may still have a negative impact on the unborn child.^15^ SSRIs block the serotonin transporter and thereby inhibit the reuptake of the neurotransmitter serotonin into the presynaptic cell. As a result, extracellular serotonin levels are increased. SSRIs can cross the placenta and are found in the amniotic fluid,^16,17^ affecting therefore not only the mother but also the developing child. Negative outcomes that have been reported include attenuated infant basal cortisol levels^18,19^ and a reduced cortisol and heart response to stressors.^20,21^ Furthermore, several behavioral changes have been reported, such as disrupted sleep patterns in newborns,^22^ and increased internalizing and externalizing behavior in toddlers.^23,24^ Recently, there has been much interest in the link between SSRI treatment and autism spectrum disorders (ASD). A meta-analysis by Andalib et al. confirms an association between the prenatal use of SSRIs and the increased risk of ASD in the child.^25^ A meta-analysis by Brown and collegues^26^ also showed that there is a link between maternal SSRI use and ASD in children; however, this effect only remained statistically significant for exposure in the first trimester when corrected for the underlying maternal mental illness. Moreover, a meta-analysis by Kaplan et al.^27^ showed that maternal depression without SSRI use also increased the risk of ASD in children. Interestingly, the risk for males to develop autism is 4 times higher compared to females.^28,29^ Remarkably, in the womb, different sex strategies take place. Compared with females, males prioritize feto-placental growth pathways at the cost of placental reserve capacity. This results in a greater risk for adverse outcomes in males especially when undernutrition or overnutrition takes place.^29,30^ Whether this also affects the adaptation capacity to SSRIs remains to be determined.

The molecular basis of the impact of SSRIs and/or the underlying depression on offspring development most likely originates from disruption of the serotonergic system.^31^ Serotonin is a key factor in regulating embryonic neuronal development.^32^ The placenta itself also synthesizes serotonin, which has been demonstrated to be essential for fetal brain development.^33^ In addition, placental synthesis of serotonin is involved in further placenta formation and decidualization of the endometrium at early stages of pregnancy.^34^ Serotonin homeostasis during pregnancy is pivotal as an imbalance might have detrimental effects on the fetus.^35^ Due to its vasoconstrictive properties,^36^ serotonin exerts a contractile response upon the placental vasculature.^37^ The resulting elevated pulse pressure is thought to be involved in the development of preeclampsia.^38^ In summary, serotonin is essential for fetal development, plays a role in placental functioning, and a significant share of serotonin for fetal brain development is synthesized by the placenta.^39,40^ However, how disturbed maternal serotonin levels affect the placenta, and the mechanisms by which this may possibly mediate immediate and long-lasting effects on the developing offspring, have yet to be established.

We previously studied the effect of SSRI exposure on placental gene expression in a microarray study, which revealed 109 differentially expressed genes in placentas of women on SSRIs.^41^ Enriched pathways included cellular growth and proliferation, cardiovascular system development and function, and inflammatory responses.^41^ Even though clear pathways were indicated in this study, placentas included were collected from women who used different types of SSRIs (all analyzed together). In addition, male and female placentas were not analyzed separately while placental gene expression is dependent on sex. In the current study, we therefore aimed to unravel the potential mechanisms by which the villous tissue of the placenta is affected after exposure to a single SSRI, namely, fluoxetine. For this, we examined placental gene expression in women with depression treated with fluoxetine during pregnancy with placental gene expression in women with a healthy pregnancy. We examined the whole transcriptome by RNA sequencing as this method can detect a higher percentage of differentially expressed genes compared to a microarray method, especially in genes with low expression.^42−44^ Our second aim of the study was to investigate whether gene expression is differently affected in placentas of boys and girls who were exposed to the SSRI fluoxetine during prenatal development and compare it to the placentas of control women. These expression profiles will give us insight into the role of the placenta in transmitting critical serotonin signals to the developing embryo, and whether male placentas are differently affected by maternal serotonin disturbances compared with female placentas.

## Results and Discussion

### Maternal Disturbances in Serotonin Levels Affect Placental Gene Expression

**Figure 1:**
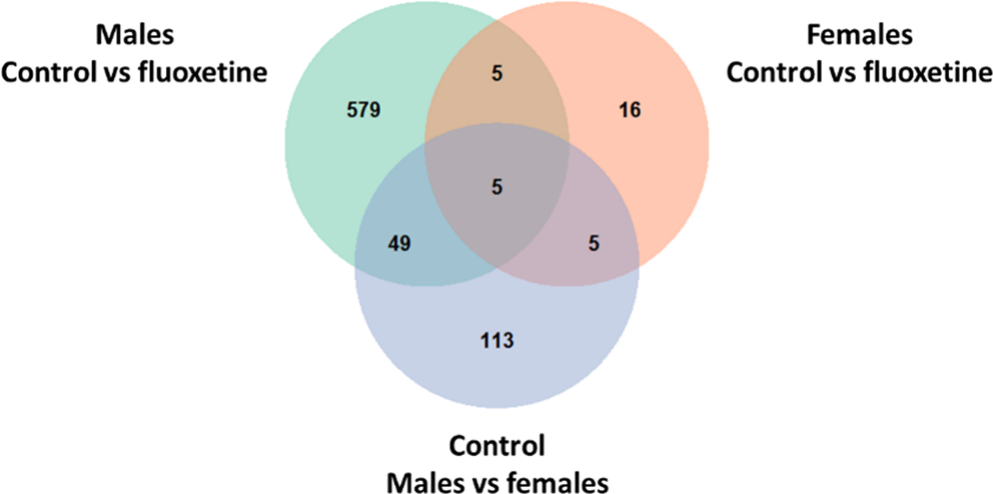
Overlap between the number of DEGs found within different models. (Top left) Number of DEGs found when comparing males in the control group and fluoxetine group. (Top right) Number of DEGs found when comparing females in the control group and fluoxetine group. (Bottom) Number of DEGs found when comparing males and females in the control group.

In this study, we aimed to elucidate how disturbances in the maternal serotonergic system affect the placenta by assessing whole transcriptomes in the placentas of women with healthy pregnancies and women treated with the SSRI fluoxetine during pregnancy. FastQC quality control was used to check the read quality. Per-base-sequence-quality and per-sequence-quality scores met the thresholds for all samples. The sequences were checked for overall GC content, which ranged between 49 and 52%. Per sample between 19.0 and 24.5 million reads were mapped with an overall alignment score of >90% for each sample, which is indicative of successful mapping to the reference genome (Supporting Information S1). Based on a principal component analysis we excluded one of the female samples in the fluoxetine group as it did not cluster with the other samples (Supporting Information S2). Male placentas prenatally exposed to fluoxetine had 638 differently expressed genes (DEGs) compared to control placentas, while for female placentas we only found 31 DEGs associated with prenatal fluoxetine exposure. The full list of DEGs and their accompanying statistics are listed in Supporting Information S4. A part of the DEGs associated with fluoxetine exposure in male and female placentas corresponds with sex-associated DEGs (Figure 1).

**Figure 2:**
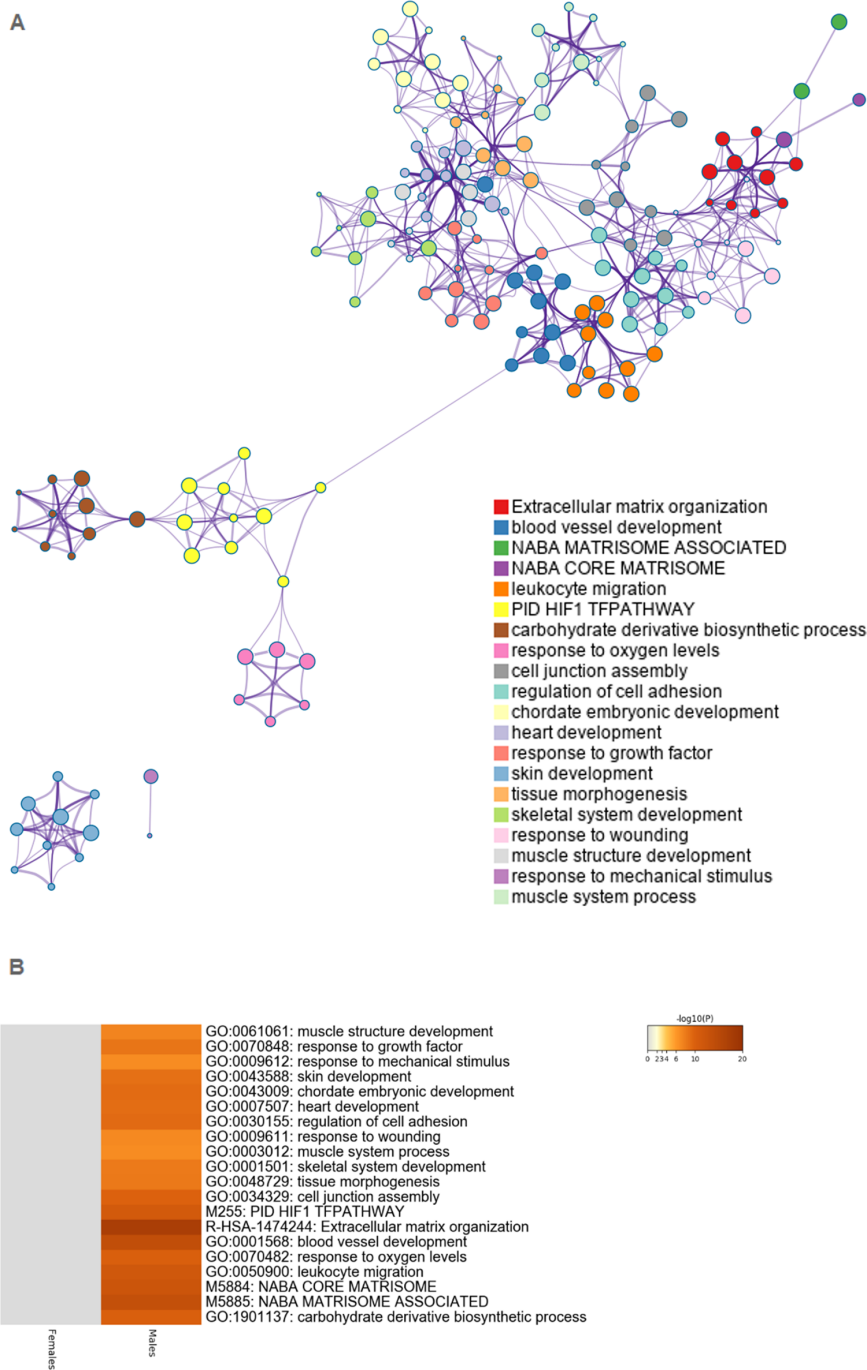
Top 20 enriched terms for DEGs in male and female placentas associated with fluoxetine treatment. (A) Network of the enriched top 20 enriched terms. Each node represents an enriched term, where its size is proportional to the number of differentially expressed genes that fall into the term. Each node is colored by its cluster ID, where nodes that share the same cluster ID are typically close to each other. Terms with a similarity score >0.3 are linked by an edge (the thickness of the edge represents the similarity score). (B) Heatmap of the top 20 enriched terms, colored by −log_10_*p*-value, the darker the color, the more statistically significant the enrichment term is. Gray cells indicate the lack of enrichment for that term in the corresponding gene list. A top 100 of enriched terms can be found in Supporting Information S7.

Functional enrichment analysis was performed to retrieve a functional profile of the differentially expressed genes in male and female placentas associated with fluoxetine treatment. This revealed several annotations that were overrepresented among the DEGs. Most notably, fluoxetine treatment affected processes of extracellular matrix organization (ECM), including the NABA matrisome associated gene set (ensemble of genes encoding ECM-associated proteins including ECM-affiliated proteins, ECM regulators and secreted factors), NABA core matrisome (ensemble of genes encoding core extracellular matrix including ECM glycoproteins, collagens, and proteoglycans), cell junction assembly, and regulation of cell adhesion. In addition, several developmental pathways including heart development, muscle structure development, chordate embryonic development, blood vessel development, skin development, and skeletal system development were significantly overrepresented in the DEGs in placentas from fluoxetine-treated mothers. Also, gene sets related to low oxygen levels, including the PID HIF1 TF pathway (HIF-1-α transcription factor network) were found to be affected (Figure 2a). Accompanying *p*-values and the contribution of male and female DEGs in the network can be found in Supporting Information S5 and S6. These functional profiles are particularly enriched in male placentas (Figure 2b). A heatmap of the top 100 enriched terms can be found in Supporting Information S7. In female placentas, the 31 DEGs included an overrepresentation of genes involved in “multicellular processes involving another multicellular organism”, inflammation responses, and response to external stimuli; most of these processes were also overrepresented in the DEGs of male placentas (Supporting Information S7).

The ECM is the noncellular component of all tissues. It not only forms the physical environment surrounding cells, it also plays structural and signaling roles, such as anchoring, guiding, or restraining cell and tissue movements.^45−49^ The ECM has been described as having a role in neural development,^50^ by regulating cell shape, proliferation, differentiation, and migration. The human placenta expresses ECM genes that can be classified into collagens, noncollagenous glycoproteins, and proteoglycans.^51^ The ECM plays an important role in placenta formation and attachment and detachment from the uterine wall.^51,52^ To our knowledge SSRIs have only been linked to the ECM once. Li et al.^53^ investigated whether fluoxetine affected the extracellular matrix in the pulmonary artery of rats with pulmonary arterial hypertension. They found that fluoxetine reduced the elastin and collagen deposition and degradation. Recent studies show that an increase in hippocampal ECM underlies memory deficits associated with prolonged stress and a depressive-like phenotype in rats.^54,55^ Other stress-related studies show a downregulation of ECM structures.^56−59^ Genes associated with the ECM that were differentially expressed in our study were mostly upregulated in placentas nourishing male offspring in pregnant women treated with fluoxetine. As changes in the ECM have previously been linked to stress, depressive-like symptoms, and SSRI treatment, it remains to be established whether the associations found in our study were the results of the SSRI treatment or the underlying depression in our subjects. It should be noted, though, that the depression scores of the mothers at weeks 17 and 32 of the pregnancy (for boy placentas in the SSRI treatment) were not indicative of ongoing depression, suggesting that the SSRI treatment had ameliorated the symptoms of the underlying depression. However, we cannot rule out that alterations in genes due to the maternal depression may still be present.

Integrins are the main transmembrane receptors that bind cells to the ECM, and their dysfunction has been linked with autism spectrum disorder (ASD).^60^ Although a bit speculative as this is a brain disorder, ASD has been highly debated as having a possible link with prenatal SSRI treatment.^25^ Still, it is of interest due to the higher prevalence of ASD in males compared to females.^28^ Integrins regulate processes associated with neural connectivity, such as neurite outgrowth and guidance, formation and maintenance of dendritic spines, and synaptic plasticity, implicating their role in nervous system development.^61−66^ We found three integrins (Integrin α2, Integrin α5, and Integrin α11) to be upregulated in male placentas exposed to fluoxetine. However, the most compelling evidence for an integrin gene association with ASD comes from studies on Integrin β3 (*itgb3*). This gene is listed by the Simon Foundation Autism Research Initiative (SFARI) as a strong candidate for association with ASD. We did not find an association with fluoxetine exposure and *itgb3* gene expression in the placenta. In addition to the link with ASD, a recent meta-analysis showed that women who receive SSRIs during pregnancy had a significantly higher risk of preterm birth compared with healthy and depressed women not on SSRIs.^67^ Interestingly, the ECM has been linked to preterm birth.^68^ The mechanical properties of cervical tissue are derived from its ECM,^69^ and abnormalities of these structures are associated with increased risk of preterm birth.^70,71^ Since we only investigated these genetic alterations in the fetal part of the placenta, we cannot conclude that the transcriptome of the maternal side of the placenta is affected in a similar way. However, fluoxetine has been shown to directly alter ECM proteins,^53^ which indicates that changes in the ECM are a potential candidate mechanism underlying the increased risk of preterm birth in women using SSRIs during pregnancy.

We also visually inspected the expression profiles of seven genes in the serotonergic pathway involved in the synthesis, transport, recognition, and degradation of serotonin, to assess whether or not these genes were affected in the placentas of women treated with fluoxetine. We did not find evidence that either *slc6a4a*, *tph1*, *tph2*, *htr1a*, *htr2a*, *htr2c*, or *maoa* were significantly altered in placental expression after SSRI treatment. This is in contrast to a previous study, that reported upregulated expression of the serotonin transporter (*sert*/*slc6a4*) in the placentas of women on SSRIs.^72^ Again, the SSRI group in this study consisted of women on several types of SSRIs, namely, sertraline, fluoxetine, citalopram, and escitalopram. Furthermore, another study including the same four types of SSRIs did not find an association with SSRI use and altered *slc6a4* expression levels in the placenta,^73^ but did find that *htr1a* gene expression and HTR1A protein levels were increased in the placenta of women with untreated depression, whereas the placentas of women on antidepressant treatment had similar expression to healthy controls. That study also found no effect for placental *maoa*, *tph1*, or *tph2* gene expression in association with SSRI exposure. To our knowledge, there is no data available on the gene expression of *htr2a* and *htr2c* in the placenta associated with SSRI exposure. SSRI exposure in our and other studies does not clearly indicate significant alterations in placental gene expression in the serotonergic pathway as a result of prenatal SSRI exposure. However, this does not mean that serotonergic pathways in the offspring itself were not affected. Gene expression patterns (in the placenta) do not always overlap with protein levels.^74^ In addition, different types of SSRIs have different pharmacokinetic properties. For example, their half-lives differ, they differentially inhibit certain enzymes and only fluoxetine has an active metabolite which is pharmacologically comparable to fluoxetine.^75^ We therefore recommend that future studies analyze the effects of different types of SSRIs separately to avoid inconsistency in results.

In our previous study, we also investigated the effect of SSRI exposure on placental gene expression in humans with a microarray and found that 109 genes were differentially expressed.^41^ We compared the 109 differentially expressed genes with our results (i.e., the 659 DEGs associated with SSRI treatment in male and/or female placentas) and found that only five genes overlapped. We suspect this might be due to the difference in study design as the previous study did not separate males from females. In the current study, we analyzed male and female placentas separately and found that the effects of SSRIs on placental gene expression were dependent on the sex of the newborn. Clustering both sexes might not reveal differences that can only be found in one of the sexes, which appears to be of major relevance based on the findings of our current study. In addition, in the current study, we looked solely at the effect of the SSRI fluoxetine, while the previous study included different types of SSRIs that were all analyzed together. The previous study included women using sertraline (*n* = 3), fluoxetine (*n* = 1), or escitalopram (*n* = 1).

### Placental Gene Expression Is Affected in a Sex-Specific Manner

**Figure 3:**
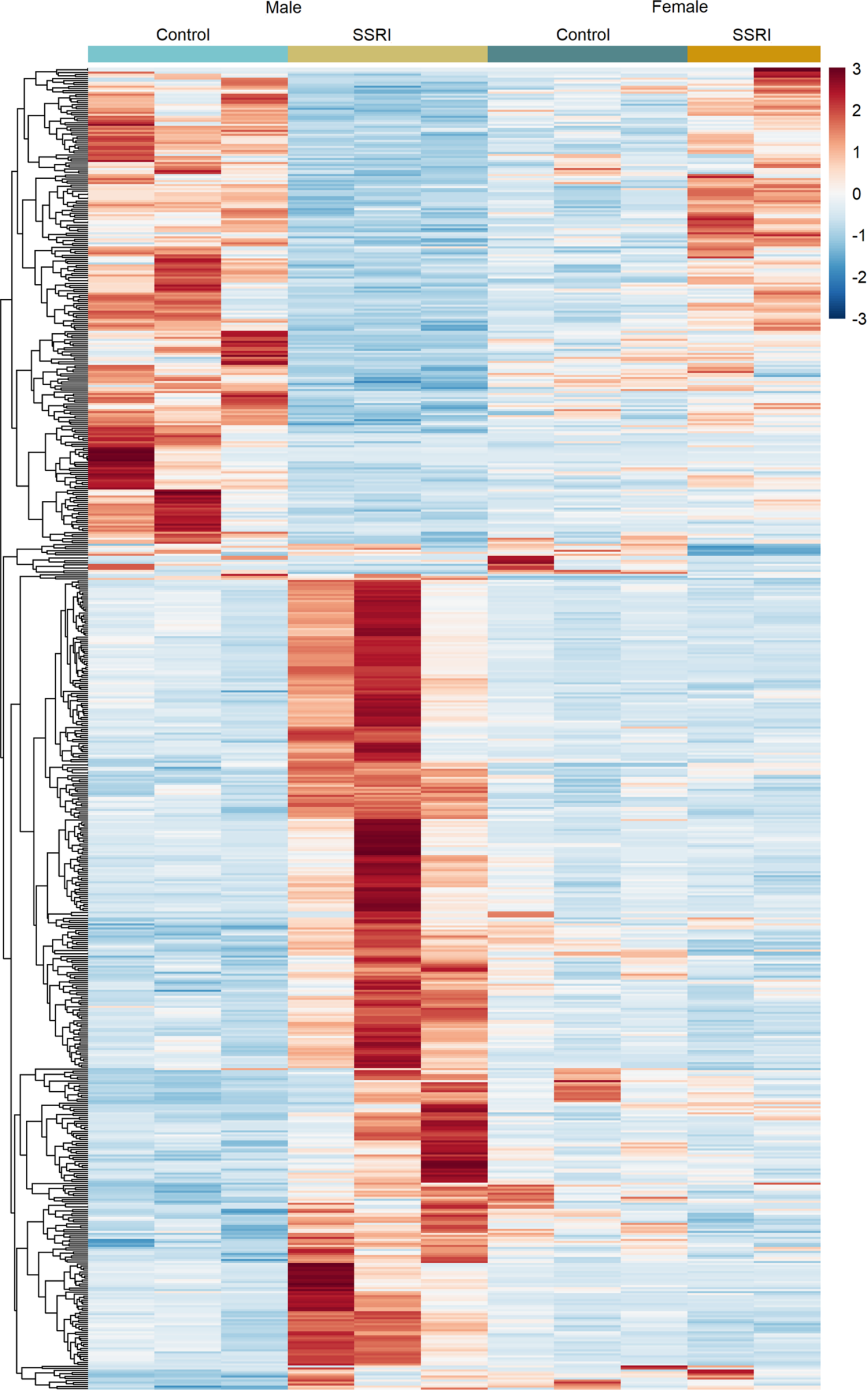
Hierarchically clustered heatmap displaying the expression patterns of DEGs found to be associated with fluoxetine treatment in male and female placentas.

We found that especially placentas nourishing male fetuses that were prenatally exposed to fluoxetine showed a distinct transcriptomic landscape (Figure 3), with 638 differentially expressed genes (DEGs). In contrast, in female placentas, we could only identify 31 genes with changed expression associated with fluoxetine exposure. This may be partly due to reduced statistical power, as we had to exclude a female sample from our analysis. In male placentas, approximately one-third of the DEGs switched from high expression in control placentas to very low expression upon SSRI treatment. For half of these DEGS expression remained similar in female placentas. Interestingly, the other half of these DEGs showed opposite patterns in female placentas, suggesting that SSRIs feminize and masculinize the gene expression patterns of these genes in the placentas of the opposite sex. The remaining two-thirds of DEGs in male placentas were strongly upregulated after SSRI treatment compared to the expression in male control placentas, while in female, the gene expression appeared insensitive to SSRIs for several hundred genes that were substantially upregulated in male placentas after maternal SSRI exposure. To determine if the genes associated with fluoxetine exposure are naturally sex-specifically expressed, we also compared the transcriptomes of healthy male and female placentas. We found that 172 genes had different expression levels in the placentas of the different sexes in healthy pregnancies (Heatmap in Supporting Information S3). Our results suggest that in male placentas nearly 10% (54 out of 638) of the DEGs associated with fluoxetine exposure are differentially expressed in males and females naturally, while for female placentas this was true for about one-third (10 out of 31) of the fluoxetine associated DEGs (Figure 1). In conclusion, gene expression in fetal placentas differs between women treated with the SSRI fluoxetine during pregnancy and healthy controls. Moreover, more genes in the placentas of women carrying male fetuses exhibited changed expression due to fluoxetine treatment compared to placentas of women carrying female fetuses. A limitation that needs to be addressed is that the sample sizes in our study, especially female placentas treated with fluoxetine, were limited. We have corrected this using false discovery rate adjustments; however, false positives and false negatives for differentially expressed genes cannot be ruled out. A small number of biological replications lowers the power and accuracy of RNaseq studies.^76^ Even though the results of the current study should be treated with caution, they do provide additional insights into gender differences. A follow-up study with a larger biological sample size is needed to replicate these results.

Regarding the underlying mechanism for this sex difference, we can merely speculate. We do know from animal studies that prenatal SSRI exposure can differentially affect male and female offspring.^77^ However, previous studies investigating the effects of prenatal stress and/or antidepressant use during pregnancy on placental gene expression do not usually differentiate between male and female placentas. Based on their initial human placenta observations, Meakin and colleagues hypothesized that males prioritize growth pathways to maximize their growth during adulthood ensuring the greatest chance for reproductive success.^78^ While this could be perceived as an evolutionary advantage, it could also render males less resilient to disturbances in the maternal environment, potentially exposing them to heightened risk when confronted with deviations, such as fluctuations in serotonergic levels beyond the norm. Although this evolutionary hypothesis is very plausible, Nugent and Bale^79^ proposed a more mechanistic explanation and hypothesized that plasticity in X-inactivation in female placentas may be an important contributor to sex differences in response to environmental perturbations during gestation. The human female placenta shows random patterns of X-inactivation,^80,81^ and the inactive X chromosome might reactivate within the placenta in response to intrauterine conditions.^82^ According to Nugent and Bale this may buffer females from detrimental conditions to a greater degree than males due to increased expression of important X-linked genes. However, we found that only 18 differentially expressed genes associated with fluoxetine exposure were X-linked (*al683813*.1, *enfb1*, *elf4*, *gabre*, *maged4*, *maged4B*, *mir503*, *pgrmc1*, *pnck*, *rab11fip1p1*, *rn7skp20*, *slc6a8*, *smim9*, *suv39h1*, *timp1*, *ttc3p1*, *z83843*.1, and *zdhhc9*) and only one (*uty*) was y-linked. Another study showing sex-dependent differences in the placenta is the study of Ceasrine et al.^83^ They showed that in male placentas the gene expression increased when maternal triglyceride levels were increased, while the opposite was seen in female placenta. When fed a high-fat diet in mice, they showed that fetal placenta and brain serotonin levels were susceptible to perinatal inflammation in males only. In addition, they showed that decreased fetal serotonin levels reflect decreased adult serotonin levels in the brain of mice exposed to a maternal high-fat diet,^83^ which highlights again the sex-specific alterations in placental gene expression due to the maternal environment. In agreement with previous studies, we found substantial differences in the placental expression of male versus female offspring, in the absence of SSRI.^84−86^ A subset of these genes also responded to SSRI treatment in placentas nourishing males and/or females, in some cases altering the expression toward the expression level seen in the opposite sex under control conditions. Future studies need to explore the possible underlying mechanisms of sex-specific effects associated with antidepressant use during pregnancy, especially the role of the placenta in this phenomenon.

In conclusion, our results provide a broad overview of genes in the villous tissue of the placenta associated with fluoxetine treatment in pregnant women compared to placentas of healthy pregnancies. Most strikingly, we see that more genes in the placenta of male infants show changed expression associated with fluoxetine treatment than in infants of female placentas, stressing the importance of sex-specific analyses. In addition, we identified ECM organization-related genes to be significantly enriched in association with fluoxetine. The ECM is linked to ASD as well as preterm birth, both of which are highly debated for their possible association with prenatal SSRI exposure. It would be interesting to examine the ECM in the offspring as well to explore the possible underlying mechanisms in the immediate and long-term outcomes associated with SSRI exposure. Another aspect that deserves further exploration is a higher-resolution study, at the level of cell types within the placenta. In our study, all cell types in the fetal placenta were pooled. Therefore, a possible decrease in expression in one cell type was masked by an increase in another cell type, small effects on expression in only one cell type were overlooked, or SSRI-responsive cells that were not included in the tissue samples were missed. For future studies, it would be interesting to assess the levels of proteins in the serotonergic pathway with the use of immunohistochemistry, as RNA expression does not necessarily overlap with protein levels.^87^ This would give a more direct insight as biological action occurs via proteins and not RNA. Furthermore, it remains to be established whether these differentially expressed genes are the result of the SSRI treatment itself, the underlying depression, or a combination of the two. Other key questions that need to be addressed are whether these differences are found in the fetus as well and how differences in these genes influence the development of the child, both in the womb as well as in the long run. In conclusion, we have shown that gene expression in the fetal placenta is altered due to fluoxetine treatment, and this effect is sex-dependent. The mechanisms that drive this sex-specific adaptation of gene expression after fluoxetine treatment remain unclear, but it is clear that the maternal environment and fetal sex play an important role in the gene expression of the placenta.

## Methods

### Subjects

The placentas used in this study were part of a previous study, conducted by Olivier et al.^41^ There, a selection of placentas was obtained from an ongoing longitudinal study on antenatal and postpartum depression: The Biology, Affect, Stress, Imaging and Cognition in Pregnancy and the Puerperium (BASIC) project.^88^ This study was conducted at the Department of Obstetrics and Gynaecology Uppsala University Hospital. All women attending routine ultrasound examination at gestational weeks 16–18 were invited to participate. Exclusion criteria were (1) inability to communicate adequately in Swedish, (2) protected identity, (3) age less than 18 years, and (4) bloodborne infectious diseases. Written informed consent was obtained from women who chose to participate in the BASIC project, and within this consent document women also specified whether or not blood and placental samples could be collected at delivery. Placental tissue was collected between April 2010 and September 2013. The study was approved by the Regional Ethics Committee, Uppsala, Sweden, and performed in accordance with relevant guidelines and regulations.

For the current study, the inclusion criteria were women of Caucasian origin, normal pregnancies (BMI in the normal range and no maternal chronic condition) and deliveries, and healthy offspring (no diagnosis and no admittance to neonatal care). Additional inclusion criteria for women on antidepressant treatment were the use of the SSRI fluoxetine during the entire pregnancy, also confirmed by serum concentrations of fluoxetine. Exclusion criteria for both groups were daily use of other prescribed drugs, alcohol use or smoking during pregnancy, any other maternal chronic condition or disease, and maternal age <18 or >42 years. Twelve placentas were selected for RNA sequencing, including six placentas from healthy pregnancies (three male and three female) and six placentas (three male and three female) from women who had detectable blood levels of the SSRI fluoxetine (Table 1). Women filled out web-based questionnaires in gestational weeks 17 and 32 including the Swedish version of the Edinburgh Postnatal Depression Scale (EPDS), which provided information on maternal depression.

**Table 1:** Placental Sample Characteristics^a^

	maternal age	gestation duration	fetal sex	EPDS week 17	EPDS week 32	fluoxetine dose (mg)	fluoxetine blood levels (nmol/L)
control group	30	40 + 2	boy	0	0		
	37	40 + 4	boy		5		
	33	40 + 4	girl	2	6		
	32	40 + 2	girl				
	26	38 + 6	boy	1	3		
	32	39 + 3	girl	5	5		
fluoxetine group	28	38 + 6	girl	4	6	20	533
	30	40 + 2	girl	4	16	20	102
	25	38 + 4	girl	20	19	40	739
	22	40 + 2	boy	9	8	unknown	439
	30	40 + 2	boy	13	2	unknown	556
	24	39 + 3	boy		6	10–20	551

a Edinburgh Postnatal Depression Scale (EPDS) was measured at gestational week 17 and gestational week 32. A score of 11 or higher usually indicated depression.^89^

### RNA iIsolation

A biopsy was taken with a 3 mm cube from the villous tissue of the placenta. Total RNA was isolated using a miRNeasy minikit (Qiagen, Hildern Germany). Tissue was lysed with QIAzol reagent (Qiagen) using a rotor-stator homogenizer (up to 33,000 rpm; Ingenieursburo CATM Zipper GmbH, type x120, Staufen, Germany) and chloroform (Sigma-Aldrich, St. Louis, MO) was added for phase separation. The rest of the procedure was performed as described in the manufacturer’s protocol. Quality and concentration of the RNA were evaluated using a NanoDrop 2000 (Thermo Scientific) and an RNA 6000 Nano Kit 2100 on a Bioanalyzer (Agilent Technologies, CA).

### Library Preparation and RNA Sequencing

Library preparation and sequencing of the RNA from the 12 placentas was done by Novogene, Hong Kong. The RNA was enriched using oligo (dT) beads. rRNA was removed with the use of the Ribo-Zero kit, leaving only the mRNA. By adding fragmentation buffer, the mRNA was randomly fragmented, then cDNA was synthesized using mRNA template and random hexamers primer. To initiate second-strand synthesis, custom second-strand synthesis buffer (Illumina), dNTPs, RNase H and DNA polymerase I (Thermo Scientific) were added. The double-stranded cDNA library was completed through size selection and PCR enrichment after a series of terminal repair, a ligation, and sequencing adaptor ligation. The quality of the libraries was assessed with Qubit 2.0 for preliminary library concentration, Agilent 2100 to test the inset size, and qPCR to quantify the library effective concentration precisely. Sequencing libraries were generated from a total amount of 3 μg RNA per sample using NEBNext Ultra RNA Library Prep Kit for Illumina (NEB) following manufacturer’s recommendations. The clustering of the index-coded samples was performed on a cBot Cluster Generation System using HiSeq PR Cluster Kit cBot-HS (Illumina) according to the manufacturer’s instructions. After cluster generation, the library preparations were sequenced on an Illumina HiSeq platform and 150 bp paired-end reads were generated.

### Quality Control and Read Mapping

Quality control of the RNA sequence data was done with FastQC (Babraham Institute). Average per-sequence-quality scores, GC content, and overrepresented sequences were checked. Subsequently, the RNA reads were trimmed with Trimmomatic.^90^ Adapter- and other Illumina-specific sequences were cut from the read. Furthermore, reads were scanned with a four-base sliding window, cutting the reads when the average quality per base dropped below 15. Then, remaining reads that had a length of 10 or fewer bases were dropped. HISAT v2.1.0 and StringTie v1.3.5 were used to perform read alignment and transcript assembly following the protocol of Pertea et al.^91^ Reads were aligned to reference genome using *Homo sapiens* GRCh38.85.

### Differential Expression Analysis

The DESeq2 R package^92^ was used to identify differentially expressed genes. We used a model with four levels: male control, male SSRI, female control, female SSRI. We used contrasts to make comparisons of expression among the following groups: Male SSRI versus Male Control, Female SSRI versus Female Control, and Male Control versus Female Control. After Benjamini–Hochberg correction, genes with *p* < 0.05 were considered to have significantly differential expression.

### Gene Set Enrichment Analysis

To assess changes in sets of related genes, a functional enrichment analysis was carried out using Metascape.^93^ For pathway and process enrichment analysis minimum overlap was set to 3 and minimum enrichment to 1.5. The significant *p*-value cutoff for enriched biological processes and pathways was set at 0.01. Enrichment analysis was performed for pathways (GO biological processes, Reactome Gene Sets, KEGG pathway, and Canonical Pathways), structural complex (CORUM), and miscellaneous (PaGenBase, Transcription Factor Targets, DisGeNET, and TRRUST). Networks were visualized using Cytoscape version 3.1.2.

## Supporting Information Available

Detailed figures and tables with information on the alignment rates (S1), gene expression data for each sample (S2), expression patterns of DEGs found to be differently expressed in male and female control samples (S3), DEGs found when comparing male and female control samples (S4a), DEGs found for the model design 4 conditions contrasted for males (S4b), DEGs found for the model design 4 conditions contrasted for females (S4c), network of enriched terms associated with fluoxetine treatment in male and female placenta (S5), network of enriched terms associated with fluoxetine treatment in male and female placenta color-coded by *p*-value (S6), and a heatmap of top 100 enriched terms across input gene list (S7) (PDF)
