# Supplementary material for: Sex-Specific Transcriptomic Changes in the Villous Tissue of Placentas of Pregnant Women Using a Selective Serotonin Reuptake Inhibitor
Source: ACS Chem Neurosci. 2024 Feb 29;15(6):1074–83. doi: 10.1021/acschemneuro.3c00621 (PMC10958514; doi:10.1021/acschemneuro.3c00621)

## Supporting Information for

# Sex-Specific Transcriptomic Changes in the Villous Tissue of Placentas of Pregnant Women using a Selective Serotonin Reuptake Inhibitor

Laura Staal[1,2], Torsten Plösch[3,4], Theodora Kunovac Kallak[6], Inger Sundström Poromaa[6], Bregje Wertheim[5#], and Jocelien D. A. Olivier[1*#]

[1]Neurobiology, Groningen Institute for Evolutionary Life Sciences, University of Groningen, 9700CC Groningen, The Netherlands

[2] Department of Cardiology, University Medical Center Groningen, University of Groningen, 9713 GZ, Groningen, the Netherlands

[3]Department of Obstetrics and Gynaecology, University Medical Center Groningen, University of Groningen, 9713 GZ Groningen, The Netherlands

[4]Department of Perinatal Neurobiology, Department of Human Medicine, School of Medicine and Health Sciences, Carl von Ossietzky University Oldenburg, 26129 Oldenburg, Germany

[5]Evolutionary Genetics, Development & Behaviour, Groningen Institute for Evolutionary Life Sciences, University of Groningen, 9700 CC Groningen, The Netherlands

[6] Department of Women's and Children's Health, Uppsala University, 75185 Uppsala, Sweden

#Authors contributed equally

* corresponding author: Jocelien Olivier; Email: j.d.a.olivier@rug.nl

### This PDF includes:

Figs and tables S1 to S7

*S1. **Alignment rates.** The total number and/or percentage of reads that could be mapped to a reference genome.*

| Sample | Input reads (100% paired) | Aligned 0 times | Aligned 1 time | Aligned > 1 times | Overall alignment |
|---|---|---|---|---|---|
| 1 | 20572927 | 1478129 (7,18%) | 17427041 (84,71%) | 16667766 (8,11%) | 95,99% |
| 2 | 19855265 | 1383382 (6,97%) | 17401035 (87,64%) | 1070848 (5,39%) | 96,21% |
| 3 | 19088262 | 1867399 (9,78%) | 15363106 (80,84%) | 1857757 (9,73%) | 94,54% |
| 4 | 20517543 | 1369028 (6,67%) | 17592798 (85,75%) | 1555717 (7,58%) | 96,34% |
| 5 | 20867264 | 1594600 (7,64%) | 18133636 (86,90%) | 1139028 (5,46%) | 95,63% |
| 6 | 23676790 | 1630498 (6,89%) | 20688449 (87,38%) | 1357843 (5,73%) | 96,29% |
| 7 | 20998362 | 1743198 (8,30%) | 18326286 (87,27%) | 928878 (4,42%) | 92,25% |
| 8 | 22637601 | 1556439 (6,88%) | 18122499 (80,05%) | 2958663 (13,07%) | 96,05% |
| 9 | 24596928 | 1526878 (6,21%) | 21541237 (87,58%) | 1528813 (6,22%) | 96,52% |
| 10 | 19824718 | 1610455 (8,12%) | 16934046 (85,42%) | 1280217 (6,46%) | 95,43% |
| 11 | 21693438 | 1436525 (6,62%) | 18874738 (87,01%) | 1382175 (6,37%) | 96,35% |
| 12 | 22749050 | 1586475 (6,97%) | 20003191 (87,93%) | 1159384 (5,10%) | 95,48% |

*S2. **Principal coordinates (PC) plot of gene expression data for each sample.** One female SSRI sample (circled) was determined to be an outlier and was removed before further analysis*

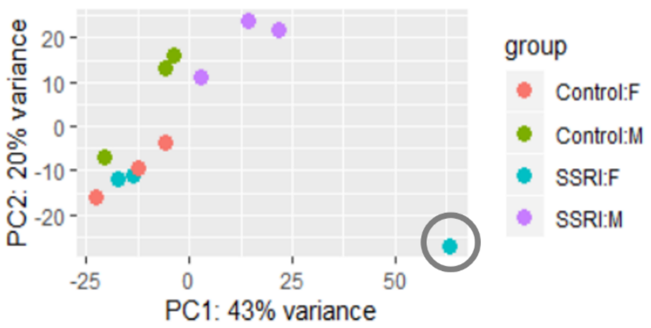

*S3. Hierarchically clustered heatmap displaying the expression patterns of DEGs found to be differentially expressed in male and female placentas in the control group.*

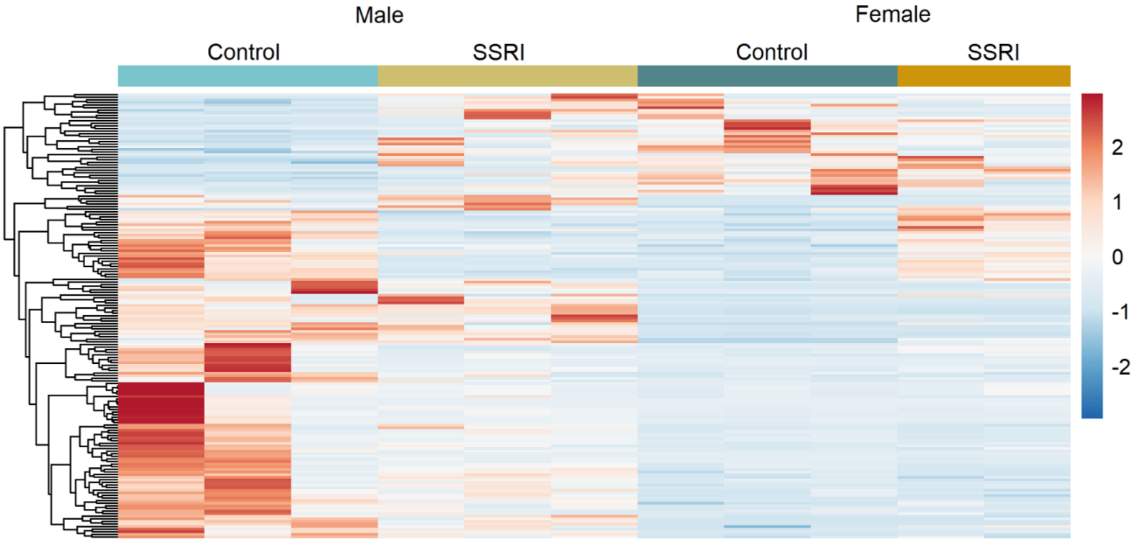


***S4a.All differentially expressed genes found comparing male control and female control samples.*** lfcSE: *standard error of the log2FoldChange.* Stat: *the Wald statistic for the Wald test, which is compared to a standard Normal distribution to generate a two-tailed* p-value. Padj: *p-value adjusted for multiple testing using the Benjamini-Hochberg method.*

| ENSEMBL ID | Gene Symbol | baseMean | log2FoldChange | lfcSE | stat | pvalue | padj |
|---|---|---|---|---|---|---|---|
| ENSG00000183878 | UTY | 624,081774 | 5,52227784 | 0,67276267 | 8,50257999 | 0,0000 | 0,0000 |
| ENSG00000169248 | CXCL11 | 135,145134 | 3,54356998 | 0,50622362 | 7,49659212 | 0,0000 | 0,0000 |
| ENSG00000176728 | TTTY14 | 72,880667 | 6,48757426 | 0,9598443 | 7,34954313 | 0,0000 | 0,0000 |
| ENSG00000275212 | AC005186.1 | 208,16019 | -4,66132657 | 0,66321847 | -7,32398822 | 0,0000 | 0,0000 |
| ENSG00000260197 | AC010889.1 | 50,8663213 | 8,78385337 | 1,4552021 | 7,19149135 | 0,0000 | 0,0000 |
| ENSG00000099715 | PCDH11Y | 154,451608 | 2,83856406 | 0,51324929 | 6,44768982 | 0,0000 | 0,0000 |
| ENSG00000179344 | HLA-DQB1 | 1577,47823 | 4,39874219 | 0,74107304 | 6,36155166 | 0,0000 | 0,0000 |
| ENSG00000196735 | HLA-DQA1 | 887,782569 | 3,47367623 | 0,62214819 | 6,28698148 | 0,0000 | 0,0000 |
| ENSG00000230663 | FAM224B | 56,0699788 | 4,90606557 | 0,84638453 | 6,21805398 | 0,0000 | 0,0000 |
| ENSG00000138755 | CXCL9 | 821,810052 | 8,56798669 | 1,84050451 | 5,95902615 | 0,0000 | 0,0000 |
| ENSG00000154451 | GBP5 | 477,009666 | 3,56536182 | 0,67608002 | 6,0012751 | 0,0000 | 0,0000 |
| ENSG00000206159 | GYG2P1 | 40,6776117 | 5,44433927 | 0,99637641 | 5,97860665 | 0,0000 | 0,0000 |
| ENSG00000099725 | PRKY | 740,045709 | 6,49768286 | 1,25745519 | 5,96400381 | 0,0000 | 0,0000 |
| ENSG00000251349 | MSANTD3-TMEFF1 | 41,7570487 | 21,4696151 | 3,85855368 | 5,8165265 | 0,0000 | 0,0000 |
| ENSG00000260342 | AC138811.2 | 295,361365 | -22,1908234 | 4,02776776 | -5,78066224 | 0,0000 | 0,0000 |
| ENSG00000067646 | ZFY | 212,082577 | 4,12416113 | 0,81702858 | 5,68409357 | 0,0000 | 0,0000 |
| ENSG00000280383 | Z95331.1 | 127,433968 | 1,91092654 | 0,40390059 | 5,58171361 | 0,0000 | 0,0000 |
| ENSG00000211677 | IGLC2 | 181,871224 | 4,28881225 | 0,87740383 | 5,54159036 | 0,0000 | 0,0000 |
| ENSG00000174697 | LEP | 17214,7911 | -5,59691322 | 1,1775973 | -5,4702067 | 0,0000 | 0,0001 |
| ENSG00000259753 | AC068234.1 | 87,4554554 | -20,8169314 | 4,23272327 | -5,44822215 | 0,0000 | 0,0001 |
| ENSG00000281490 | CICP14 | 1472,81349 | 1,36596725 | 0,27034481 | 5,4545382 | 0,0000 | 0,0001 |
| ENSG00000203709 | MIR29B2CHG | 2909,06969 | 1,15437136 | 0,22471666 | 5,4070678 | 0,0000 | 0,0001 |
| ENSG00000273373 | AL355488.1 | 470,629645 | 1,19369886 | 0,23299447 | 5,41465899 | 0,0000 | 0,0001 |
| ENSG00000169245 | CXCL10 | 376,753762 | 3,98402464 | 0,86043619 | 5,40985608 | 0,0000 | 0,0001 |
| ENSG00000186148 | AC013268.1 | 1057,94065 | -2,1174545 | 0,48799482 | -5,37012336 | 0,0000 | 0,0001 |
| ENSG00000146250 | PRSS35 | 167,337885 | -2,73309501 | 0,65927814 | -5,30891889 | 0,0000 | 0,0001 |
| ENSG00000244116 | IGKV2-28 | 56,628278 | 6,27361899 | 1,4776134 | 5,29094959 | 0,0000 | 0,0001 |
| ENSG00000149346 | SLX4IP | 437,019152 | 1,14423686 | 0,22871972 | 5,2835061 | 0,0000 | 0,0001 |
| ENSG00000154620 | TMSB4Y | 24,5064953 | 6,53014267 | 1,5960477 | 5,25839197 | 0,0000 | 0,0001 |
| ENSG00000131002 | TXLNGY | 324,937801 | 6,15512695 | 1,49694551 | 5,20515122 | 0,0000 | 0,0002 |
| ENSG00000242534 | IGKV2D-28 | 55,9221842 | 7,01471181 | 1,84620064 | 5,19678316 | 0,0000 | 0,0002 |
| ENSG00000198692 | EIF1AY | 608,680907 | 5,05495254 | 1,19004879 | 5,13846984 | 0,0000 | 0,0002 |
| ENSG00000179583 | CIITA | 1152,0204 | 2,96763352 | 0,75356451 | 5,13469243 | 0,0000 | 0,0002 |
| ENSG00000233522 | FAM224A | 36,8321667 | 4,26706909 | 1,04864682 | 5,04955168 | 0,0000 | 0,0003 |

| | | | | | | | |
|---|---|---|---|---|---|---|---|
| ENSG00000273003 | ARL2-SNX15 | 26,3123054 | -18,3302285 | 5,44118198 | -4,99018598 | 0,0000 | 0,0004 |
| ENSG00000172116 | CD8B | 16,8842718 | 6,65922924 | 1,98840616 | 4,97509414 | 0,0000 | 0,0005 |
| ENSG00000249267 | LINC00939 | 325,522 | -3,78071924 | 0,9870006 | -4,96646449 | 0,0000 | 0,0005 |
| ENSG00000012817 | KDM5D | 1592,16323 | 4,61965866 | 1,22633317 | 4,91278606 | 0,0000 | 0,0006 |
| ENSG00000267904 | AC024075.1 | 21,9153032 | -17,5506563 | 5,94826047 | -4,89131592 | 0,0000 | 0,0007 |
| ENSG00000278212 | AC134878.2 | 16,2145988 | 6,27068822 | 1,98849999 | 4,86719653 | 0,0000 | 0,0007 |
| ENSG00000090104 | RGS1 | 760,880143 | 2,55613979 | 0,75164724 | 4,80961437 | 0,0000 | 0,0010 |
| ENSG00000116690 | PRG4 | 27,2120674 | 4,33966216 | 1,24492836 | 4,79910896 | 0,0000 | 0,0010 |
| ENSG00000204252 | HLA-DOA | 354,372613 | 2,45990603 | 0,72487504 | 4,79646335 | 0,0000 | 0,0010 |
| ENSG00000234290 | AC116366.1 | 238,302305 | 1,32156906 | 0,31326356 | 4,7810083 | 0,0000 | 0,0010 |
| ENSG00000256276 | | 66,8588668 | -3,60631001 | 1,06825664 | -4,76650821 | 0,0000 | 0,0011 |
| ENSG00000204287 | HLA-DRA | 8618,14081 | 2,00597227 | 0,56796634 | 4,76058836 | 0,0000 | 0,0011 |
| ENSG00000231535 | LINC00278 | 16,4300272 | 5,86869513 | 2,10346785 | 4,7193372 | 0,0000 | 0,0013 |
| ENSG00000239218 | RPS20P22 | 38,1825387 | 3,02113716 | 0,95148629 | 4,69829328 | 0,0000 | 0,0014 |
| ENSG00000140749 | IGSF6 | 530,581447 | 0,93202559 | 0,21690286 | 4,69703048 | 0,0000 | 0,0014 |
| ENSG00000231389 | HLA-DPA1 | 3611,73969 | 2,06909976 | 0,61749978 | 4,68559356 | 0,0000 | 0,0014 |
| ENSG00000187094 | CCK | 1022,49007 | -2,8851301 | 0,94608913 | -4,64050873 | 0,0000 | 0,0017 |
| ENSG00000158055 | GRHL3 | 172,895531 | -1,39881468 | 0,35779931 | -4,64189972 | 0,0000 | 0,0017 |
| ENSG00000121005 | CRISPLD1 | 329,996206 | -2,79934339 | 0,93871572 | -4,60804395 | 0,0000 | 0,0020 |
| ENSG00000242574 | HLA-DMB | 1539,6089 | 1,36085357 | 0,35621496 | 4,57539451 | 0,0000 | 0,0023 |
| ENSG00000253196 | AC083841.1 | 13,193454 | 4,92160158 | 2,07485053 | 4,50713012 | 0,0000 | 0,0031 |
| ENSG00000103044 | HAS3 | 381,450762 | -2,00020446 | 0,67726422 | -4,47520639 | 0,0000 | 0,0035 |
| ENSG00000211592 | IGKC | 542,753034 | 3,75481241 | 1,6086993 | 4,38969757 | 0,0000 | 0,0051 |
| ENSG00000280153 | AC133065.3 | 158,233133 | 1,85132668 | 0,64544098 | 4,38912437 | 0,0000 | 0,0051 |
| ENSG00000164308 | ERAP2 | 1495,58808 | -1,95682126 | 0,71305536 | -4,37542621 | 0,0000 | 0,0052 |
| ENSG00000229807 | XIST | 18934,8646 | -3,71369711 | 1,61744936 | -4,37502042 | 0,0000 | 0,0052 |
| ENSG00000223534 | HLA-DQB1-AS1 | 14,2593398 | 5,00960417 | 2,89680992 | 4,37330913 | 0,0000 | 0,0052 |
| ENSG00000243302 | AC018638.4 | 1058,10114 | 1,20333331 | 0,34186622 | 4,36846775 | 0,0000 | 0,0052 |
| ENSG00000279312 | AL136164.4 | 695,253207 | 1,26469739 | 0,3706995 | 4,34585227 | 0,0000 | 0,0057 |
| ENSG00000197249 | SERPINA1 | 1149,57113 | 1,49064302 | 0,47612802 | 4,33444908 | 0,0000 | 0,0059 |
| ENSG00000211679 | IGLC3 | 108,941708 | 3,01283668 | 1,35582174 | 4,31042056 | 0,0000 | 0,0065 |
| ENSG00000279162 | AC141586.5 | 62,6315671 | 1,4925935 | 0,48985473 | 4,3032995 | 0,0000 | 0,0066 |
| ENSG00000233070 | ZFY-AS1 | 25,7712439 | 3,6019553 | 1,78775955 | 4,27886952 | 0,0000 | 0,0070 |
| ENSG00000168955 | TM4SF20 | 19,263033 | 4,75292015 | 3,3914472 | 4,28029864 | 0,0000 | 0,0070 |
| ENSG00000188676 | IDO2 | 4302,16307 | 1,38636018 | 0,44482434 | 4,28087691 | 0,0000 | 0,0070 |
| ENSG00000165246 | NLGN4Y | 45,8405987 | 2,78827995 | 1,28412245 | 4,27761322 | 0,0000 | 0,0070 |
| ENSG00000258653 | AC005520.1 | 28,3048855 | 4,12144804 | 2,32762665 | 4,268852 | 0,0000 | 0,0072 |
| ENSG00000180875 | GREM2 | 1158,01232 | -1,30763378 | 0,4143827 | -4,26161644 | 0,0000 | 0,0073 |
| ENSG00000127366 | TAS2R5 | 64,0684438 | 1,80192894 | 0,73119325 | 4,22047882 | 0,0000 | 0,0087 |
| ENSG00000227097 | RPS28P7 | 4063,10841 | -2,01564525 | 0,88639615 | -4,21331529 | 0,0000 | 0,0088 |
| ENSG00000253939 | AC007991.3 | 64,9028815 | 1,85147931 | 0,78164442 | 4,20262208 | 0,0000 | 0,0091 |
| ENSG00000271533 | Z83843.1 | 946,6827 | 1,04435759 | 0,32708455 | 4,19159039 | 0,0000 | 0,0094 |
| ENSG00000152766 | ANKRD22 | 53,8764898 | 3,72565914 | 2,33119594 | 4,17762584 | 0,0000 | 0,0099 |
| ENSG00000123999 | INHA | 3060,4934 | -1,50546042 | 0,57034494 | -4,16352046 | 0,0000 | 0,0104 |
| ENSG00000241475 | AL160408.4 | 19,84813 | -3,30544214 | 1,97479109 | -4,14678412 | 0,0000 | 0,0111 |
| ENSG00000117091 | CD48 | 210,851567 | 1,42472503 | 0,53359549 | 4,13825972 | 0,0000 | 0,0111 |
| ENSG00000279759 | AC118344.2 | 330,026039 | 1,26964126 | 0,44230267 | 4,14166025 | 0,0000 | 0,0111 |
| ENSG00000090382 | LYZ | 2744,31236 | 1,46869037 | 0,56225058 | 4,13809826 | 0,0000 | 0,0111 |
| ENSG00000255197 | AC090559.1 | 122,495619 | 1,55275308 | 0,62352192 | 4,1336824 | 0,0000 | 0,0111 |
| ENSG00000229308 | AC010737.1 | 25,5814374 | 3,78599505 | 3,07556386 | 4,12551052 | 0,0000 | 0,0113 |
| ENSG00000280800 | FP671120.3 | 1959,12438 | 2,10819503 | 1,06134547 | 4,1280259 | 0,0000 | 0,0113 |
| ENSG00000259884 | AC025259.3 | 24,7713879 | 2,2488025 | 1,18159353 | 4,12067421 | 0,0000 | 0,0114 |
| ENSG00000171345 | KRT19 | 47558,528 | -1,12189966 | 0,38195808 | -4,11417947 | 0,0000 | 0,0116 |
| ENSG00000281383 | FP671120.4 | 1082,73606 | 2,07883119 | 1,07542618 | 4,10314181 | 0,0000 | 0,0120 |
| ENSG00000158473 | CD1D | 63,1904238 | 1,79500428 | 0,8594621 | 4,08855079 | 0,0000 | 0,0126 |
| ENSG00000167604 | NFKBID | 225,85677 | 1,18970449 | 0,42790239 | 4,07975824 | 0,0000 | 0,0130 |
| ENSG00000274008 | RF00017 | 30,0999274 | 1,97693584 | 1,05599938 | 4,06063576 | 0,0000 | 0,0139 |
| ENSG00000198848 | CES1 | 291,451184 | -1,3127655 | 0,52672195 | -4,03036906 | 0,0001 | 0,0155 |
| ENSG00000281181 | FP236383.3 | 54358,5148 | 1,94329439 | 1,07118232 | 4,03285844 | 0,0001 | 0,0155 |
| ENSG00000223865 | HLA-DPB1 | 2053,91374 | 1,6128807 | 0,77174046 | 4,02746055 | 0,0001 | 0,0155 |
| ENSG00000183307 | TMEM121B | 90,4665691 | 1,24768889 | 0,4918259 | 4,01810449 | 0,0001 | 0,0160 |
| ENSG00000229951 | AC104695.2 | 64,9932957 | 1,42584481 | 0,62113141 | 4,01605275 | 0,0001 | 0,0160 |
| ENSG00000188321 | ZNF559 | 238,782345 | 1,27328176 | 0,52044098 | 3,99862117 | 0,0001 | 0,0170 |
| ENSG00000248593 | DSTNP2 | 71,1842495 | -1,48410386 | 0,69107144 | -3,99442889 | 0,0001 | 0,0171 |
| ENSG00000147571 | CRH | 48011,2869 | -2,00523939 | 1,22150769 | -3,98427043 | 0,0001 | 0,0177 |
| ENSG00000019582 | CD74 | 17474,3097 | 1,46241263 | 0,69268906 | 3,97397045 | 0,0001 | 0,0182 |
| ENSG00000114374 | USP9Y | 942,842744 | 2,80316625 | 2,17542881 | 3,9725401 | 0,0001 | 0,0182 |
| ENSG00000026751 | SLAMF7 | 164,766656 | 2,27323134 | 1,53619967 | 3,96691742 | 0,0001 | 0,0185 |
| ENSG00000121807 | CCR2 | 86,4091596 | 1,79203172 | 1,05378784 | 3,9573414 | 0,0001 | 0,0191 |
| ENSG00000196126 | HLA-DRB1 | 4366,72539 | 1,72230283 | 0,99142808 | 3,95026855 | 0,0001 | 0,0194 |
| ENSG00000265206 | AC004687.1 | 134,047927 | 1,49818811 | 0,75883931 | 3,94659441 | 0,0001 | |

| | | | | | | | |
|---|---|---|---|---|---|---|---|
| ENSG00000280614 | FP236383.2 | 10255,0434 | 1,80398542 | 1,09021524 | 3,94490281 | 0,0001 | 0,0195 |
| ENSG00000161929 | SCIMP | 227,558386 | 1,29184642 | 0,57807266 | 3,93806768 | 0,0001 | 0,0199 |
| ENSG00000178773 | CPNE7 | 61,1784868 | 2,6890075 | 2,37624061 | 3,91820751 | 0,0001 | 0,0212 |
| ENSG00000261326 | LINC01355 | 1031,34719 | 0,94002659 | 0,37408921 | 3,9186921 | 0,0001 | 0,0212 |
| ENSG00000126759 | CFP | 351,556379 | 1,40170213 | 0,70851421 | 3,90719701 | 0,0001 | 0,0220 |
| ENSG00000110848 | CD69 | 122,642735 | 1,24988483 | 0,58067721 | 3,89365334 | 0,0001 | 0,0230 |
| ENSG00000215481 | BCRP3 | 22,6360548 | -2,3625038 | 1,93026666 | -3,88830753 | 0,0001 | 0,0233 |
| ENSG00000278847 | AC006157.1 | 8,05015254 | 2,59167159 | 2,77464579 | 3,88366293 | 0,0001 | 0,0236 |
| ENSG00000163131 | CTSS | 3384,59944 | 1,17773509 | 0,5343443 | 3,87976715 | 0,0001 | 0,0238 |
| ENSG00000185686 | PRAME | 106,600549 | -2,24394176 | 1,8110992 | -3,87563649 | 0,0001 | 0,0240 |
| ENSG00000259972 | AC009120.2 | 371,974094 | 1,11138415 | 0,50232868 | 3,85675629 | 0,0001 | 0,0257 |
| ENSG00000127377 | CRYGN | 72,0074954 | -1,49598931 | 0,96944432 | -3,81550052 | 0,0001 | 0,0301 |
| ENSG00000279649 | AC020978.9 | 202,100299 | 1,14342167 | 0,55917399 | 3,81352596 | 0,0001 | 0,0301 |
| ENSG00000225783 | MIAT | 103,808323 | 1,56685492 | 1,08180196 | 3,81054507 | 0,0001 | 0,0301 |
| ENSG00000169442 | CD52 | 184,17829 | 1,18898031 | 0,60350583 | 3,80926006 | 0,0001 | 0,0301 |
| ENSG00000235568 | NFAM1 | 741,775222 | 1,09516624 | 0,52830482 | 3,80345671 | 0,0001 | 0,0305 |
| ENSG00000108187 | PBLD | 377,115095 | 0,77624919 | 0,3516818 | 3,78899509 | 0,0002 | 0,0321 |
| ENSG00000258643 | BCL2L2-PABPN1 | 58,154651 | -1,91915079 | 1,74560388 | -3,77341011 | 0,0002 | 0,0339 |
| ENSG00000261760 | AC140479.4 | 279,393504 | -1,40279427 | 0,93346254 | -3,76807447 | 0,0002 | 0,0344 |
| ENSG00000211751 | TRBC1 | 72,5393435 | 1,37167402 | 0,89708628 | 3,76273728 | 0,0002 | 0,0344 |
| ENSG00000229642 | AC087857.1 | 25,6495925 | -2,05700705 | 2,5030876 | -3,75222346 | 0,0002 | 0,0344 |
| ENSG00000064886 | CHI3L2 | 346,092556 | 1,26808785 | 0,75930408 | 3,75608076 | 0,0002 | 0,0344 |
| ENSG00000173674 | EIF1AX | 3627,11169 | -0,53864821 | 0,2705799 | -3,75476172 | 0,0002 | 0,0344 |
| ENSG00000279801 | AC111170.3 | 21,9576419 | 1,59452704 | 1,25928388 | 3,76046951 | 0,0002 | 0,0344 |
| ENSG00000134709 | HOOK1 | 306,487282 | -1,5085923 | 1,14213803 | -3,7524709 | 0,0002 | 0,0344 |
| ENSG00000279281 | AC015883.1 | 12,5156691 | 2,09730293 | 2,45003066 | 3,76110859 | 0,0002 | 0,0344 |
| ENSG00000235308 | AL445991.1 | 11,3774813 | 1,92239836 | 1,83952957 | 3,75600259 | 0,0002 | 0,0344 |
| ENSG00000140853 | NLRC5 | 895,052675 | 0,99885437 | 0,50050442 | 3,7419529 | 0,0002 | 0,0356 |
| ENSG00000131435 | PDLIM4 | 1637,87795 | -1,00201169 | 0,50712471 | -3,73589729 | 0,0002 | 0,0360 |
| ENSG00000260261 | AC124944.3 | 166,453404 | 0,86378558 | 0,41853211 | 3,7348534 | 0,0002 | 0,0360 |
| ENSG00000259365 | AC019254.2 | 9,09638557 | 1,95088432 | 2,57791658 | 3,7307552 | 0,0002 | 0,0364 |
| ENSG00000238121 | LINC00426 | 12,7470174 | 1,84694725 | 2,79534814 | 3,72689419 | 0,0002 | 0,0367 |
| ENSG00000277734 | TRAC | 84,3692333 | 1,69714844 | 1,56112273 | 3,72461707 | 0,0002 | 0,0367 |
| ENSG00000218537 | MIF-AS1 | 1949,34257 | -0,90190448 | 0,45012191 | -3,71805252 | 0,0002 | 0,0374 |
| ENSG00000142512 | SIGLEC10 | 291,445463 | 1,43000625 | 1,13851673 | 3,70686184 | 0,0002 | 0,0388 |
| ENSG00000125730 | C3 | 2451,87447 | 1,30328904 | 0,93298259 | 3,69682478 | 0,0002 | 0,0398 |
| ENSG00000272983 | AL117339.4 | 38,7481603 | 1,22328999 | 0,79489759 | 3,69792851 | 0,0002 | 0,0398 |
| ENSG00000256282 | AC112694.1 | 24,9294057 | 1,42130964 | 1,16490849 | 3,69324837 | 0,0002 | 0,0398 |
| ENSG00000183230 | CTNNA3 | 9,81534659 | -1,82722976 | 2,4727949 | -3,69402732 | 0,0002 | 0,0398 |
| ENSG00000234814 | SVILP1 | 16,1043821 | 1,79372379 | 2,50524725 | 3,68850203 | 0,0002 | 0,0403 |
| ENSG00000171174 | RBKS | 149,221037 | 1,31033428 | 0,97783294 | 3,68455423 | 0,0002 | 0,0406 |
| ENSG00000140379 | BCL2A1 | 174,942873 | 1,18932209 | 0,76997399 | 3,68277472 | 0,0002 | 0,0407 |
| ENSG00000198794 | SCAMP5 | 167,610184 | 1,01668002 | 0,56473755 | 3,67957219 | 0,0002 | 0,0409 |
| ENSG00000186818 | LILRB4 | 541,986191 | 1,0332726 | 0,58535891 | 3,67458453 | 0,0002 | 0,0414 |
| ENSG00000114646 | CSPG5 | 136,38567 | -1,18637184 | 0,7893689 | -3,66989851 | 0,0002 | 0,0419 |
| ENSG00000111432 | FZD10 | 1010,32201 | -0,9245402 | 0,50231203 | -3,66101236 | 0,0003 | 0,0431 |
| ENSG00000250138 | AC139495.3 | 480,312885 | 1,18785457 | 0,81268298 | 3,65955439 | 0,0003 | 0,0431 |
| ENSG00000180539 | C9orf139 | 75,3662463 | 1,26026842 | 0,95794311 | 3,65615007 | 0,0003 | 0,0433 |
| ENSG00000185198 | PRSS57 | 6,57808592 | 1,71184236 | 2,28767586 | 3,65329463 | 0,0003 | 0,0433 |
| ENSG00000153563 | CD8A | 132,30226 | 1,39165731 | 1,24130381 | 3,65389286 | 0,0003 | 0,0433 |
| ENSG00000283236 | AC074141.1 | 70,4275466 | 1,11635783 | 0,71619566 | 3,64990489 | 0,0003 | 0,0433 |
| ENSG00000119715 | ESRRB | 42,8279705 | 1,25629298 | 0,96758441 | 3,64989444 | 0,0003 | 0,0433 |
| ENSG00000260447 | AC009065.3 | 8,7027056 | 1,64835721 | 1,90802206 | 3,6441747 | 0,0003 | 0,0440 |
| ENSG00000197576 | HOXA4 | 9,79820489 | 1,67388487 | 2,26089799 | 3,641471 | 0,0003 | 0,0442 |
| ENSG00000213077 | | 43,8724676 | 1,35286604 | 1,21349782 | 3,63884259 | 0,0003 | 0,0443 |
| ENSG00000231739 | GAPDHP59 | 8,36267496 | 1,60587809 | 2,4151484 | 3,63528165 | 0,0003 | 0,0447 |
| ENSG00000160593 | JAML | 539,065273 | 1,04638982 | 0,64630688 | 3,63300051 | 0,0003 | 0,0448 |
| ENSG00000262319 | AC007952.6 | 121,893391 | 1,12841762 | 0,77562018 | 3,62733006 | 0,0003 | 0,0455 |
| ENSG00000211943 | IGHV3-15 | 9,94119553 | 1,48503199 | 2,51457857 | 3,62013499 | 0,0003 | 0,0465 |
| ENSG00000277632 | CCL3 | 420,49916 | 1,06900446 | 0,70246281 | 3,61495073 | 0,0003 | 0,0469 |
| ENSG00000101057 | MYBL2 | 480,299482 | -0,58350676 | 0,32883557 | -3,61540018 | 0,0003 | 0,0469 |
| ENSG00000162849 | KIF26B | 341,499123 | -1,05437171 | 0,69398576 | -3,60665711 | 0,0003 | 0,0478 |
| ENSG00000100302 | RASD2 | 22,5798762 | 1,26660002 | 1,13269846 | 3,60746146 | 0,0003 | 0,0478 |
| ENSG00000229915 | AC016999.1 | 7,44125281 | 1,5061458 | 2,27766662 | 3,59618996 | 0,0003 | 0,0494 |
| ENSG00000255571 | MIR9-3HG | 202,604666 | -1,15286205 | 0,90479849 | -3,59424495 | 0,0003 | 0,0494 |
| ENSG00000198886 | MT-ND4 | 513087,615 | -0,93118106 | 0,56283984 | -3,59358401 | 0,0003 | 0,0494 |
| ENSG00000188282 | RUFY4 | 33,7826645 | 1,52252788 | 2,0659854 | 3,59050398 | 0,0003 | 0,0497 |

*S4b. All differentially expressed genes found for the model design 4 conditions (male control, male SSRI, female control, and female SSRI), contrasted for males, comparing male control, and male fluoxetine.* lfcSE*: standard error of the log2FoldChange.* Stat*: the Wald statistic for the Wald test, which is compared to a standard Normal distribution to generate a two-tailed* p-value. Padj*: p-value adjusted for multiple testing using the Benjamini-Hochberg method.*

| ENSEMBL ID | Gene Symbol | baseMean | log2FoldChange | lfcSE | stat | pvalue | padj |
|---|---|---|---|---|---|---|---|
| ENSG00000171067 | C11orf24 | 1062.31595 | 2.11933874 | 0.20191905 | 10.4959819 | 0.0000 | 0.0000 |
| ENSG00000160161 | CILP2 | 194.609918 | 4.62977213 | 0.56063855 | 8.25803391 | 0.0000 | 0.0000 |
| ENSG00000203709 | MIR29B2CHG | 2909.06969 | -1.85928619 | 0.22974244 | -8.09291575 | 0.0000 | 0.0000 |
| ENSG00000275212 | AC005186.1 | 208.16019 | 4.75129492 | 0.66837213 | 7.10875677 | 0.0000 | 0.0000 |
| ENSG00000279312 | AL136164.4 | 695.253207 | -2.26426347 | 0.34350461 | -6.59165375 | 0.0000 | 0.0000 |
| ENSG00000273373 | AL355488.1 | 470.629645 | -1.51479753 | 0.23892684 | -6.34000573 | 0.0000 | 0.0000 |
| ENSG00000281490 | CICP14 | 1472.81349 | -1.74084727 | 0.27503051 | -6.32965144 | 0.0000 | 0.0000 |
| ENSG00000226145 | KRT16P6 | 1670.73579 | 4.86631378 | 0.77580868 | 6.27256936 | 0.0000 | 0.0000 |
| ENSG00000143641 | GALNT2 | 10003.5762 | 1.52108062 | 0.24343623 | 6.24837414 | 0.0000 | 0.0000 |
| ENSG00000171345 | KRT19 | 47558.528 | 2.028487 | 0.32577007 | 6.22674456 | 0.0000 | 0.0000 |
| ENSG00000249267 | LINC00939 | 325.522 | 5.26982356 | 0.85990933 | 6.12834794 | 0.0000 | 0.0000 |
| ENSG00000254870 | ATP6V1G2-DDX39B | 318.276138 | -24.6821658 | 4.08061339 | -6.04864107 | 0.0000 | 0.0000 |
| ENSG00000260342 | AC138811.2 | 295.361365 | 24.5225426 | 4.08055105 | 6.00961545 | 0.0000 | 0.0000 |
| ENSG00000103740 | ACSBG1 | 179.176474 | -3.99158305 | 0.67012988 | -5.95643194 | 0.0000 | 0.0000 |
| ENSG00000180530 | NRIP1 | 3237.36361 | 2.19878094 | 0.37183569 | 5.91331327 | 0.0000 | 0.0000 |
| ENSG00000248593 | DSTNP2 | 71.1842495 | 3.02716938 | 0.5161639 | 5.8647445 | 0.0000 | 0.0000 |
| ENSG00000279393 | AL139005.1 | 897.41511 | 3.02948673 | 0.52029093 | 5.82267838 | 0.0000 | 0.0000 |
| ENSG00000152952 | PLOD2 | 5080.44558 | 2.40947453 | 0.41413606 | 5.81807473 | 0.0000 | 0.0000 |
| ENSG00000221955 | SLC12A8 | 634.230794 | 3.4410392 | 0.59319654 | 5.80084167 | 0.0000 | 0.0000 |
| ENSG00000227097 | RPS28P7 | 4063.10841 | 3.74595458 | 0.64596939 | 5.79896612 | 0.0000 | 0.0000 |
| ENSG00000102755 | FLT1 | 96571.3723 | 2.30794008 | 0.39939686 | 5.77856337 | 0.0000 | 0.0000 |
| ENSG00000237330 | RNF223 | 463.739483 | 3.07194446 | 0.53357294 | 5.75730929 | 0.0000 | 0.0000 |
| ENSG00000251357 | AP000350.4 | 25.9173057 | -23.3998124 | 4.08310251 | -5.73089025 | 0.0000 | 0.0000 |
| ENSG00000271533 | Z83843.1 | 946.6827 | -1.63329102 | 0.28801184 | -5.67091627 | 0.0000 | 0.0000 |
| ENSG00000270605 | AL353622.1 | 134.958081 | -1.9334914 | 0.34702518 | -5.57161708 | 0.0000 | 0.0000 |
| ENSG00000197614 | MFAP5 | 8743.64947 | 2.0601016 | 0.3762395 | 5.47550596 | 0.0000 | 0.0000 |
| ENSG00000167880 | EVPL | 590.614516 | 2.80229116 | 0.51492534 | 5.442131 | 0.0000 | 0.0000 |
| ENSG00000213859 | KCTD11 | 1395.10567 | 1.45493838 | 0.26798312 | 5.42921644 | 0.0000 | 0.0001 |
| ENSG00000188676 | IDO2 | 4302.16307 | -2.13053024 | 0.39525732 | -5.39023607 | 0.0000 | 0.0001 |
| ENSG00000268439 | EMG1 | 88.9219421 | -2.21843682 | 0.42042433 | -5.27666131 | 0.0000 | 0.0001 |
| ENSG00000244116 | IGKV2-28 | 56.628278 | -8.3348299 | 1.57920695 | -5.27785791 | 0.0000 | 0.0001 |
| ENSG00000104332 | SFRP1 | 3983.385 | 2.74040912 | 0.52037621 | 5.26620757 | 0.0000 | 0.0001 |
| ENSG00000169248 | CXCL11 | 135.145134 | -2.50570109 | 0.47649574 | -5.25860126 | 0.0000 | 0.0001 |
| ENSG00000229642 | AC087857.1 | 25.6495925 | 8.46213863 | 1.62362956 | 5.21186533 | 0.0000 | 0.0001 |
| ENSG00000159516 | SPRR2G | 100.874428 | 5.572194 | 1.06862674 | 5.21435015 | 0.0000 | 0.0001 |
| ENSG00000136002 | ARHGEF4 | 727.092883 | 3.96312509 | 0.76360369 | 5.19002875 | 0.0000 | 0.0001 |
| ENSG00000142623 | PADI1 | 718.19109 | 3.50207069 | 0.67716418 | 5.17167153 | 0.0000 | 0.0002 |
| ENSG00000151023 | ENKUR | 64.10338 | 3.49713967 | 0.67700097 | 5.16563464 | 0.0000 | 0.0002 |
| ENSG00000242534 | IGKV2D-28 | 55.9221842 | -8.31472746 | 1.61089549 | -5.16155613 | 0.0000 | 0.0002 |
| ENSG00000232931 | LINC00342 | 282.776851 | -2.23197325 | 0.43319777 | -5.15231939 | 0.0000 | 0.0002 |
| ENSG00000135245 | HILPDA | 2833.86346 | 2.57169833 | 0.50014559 | 5.14189943 | 0.0000 | 0.0002 |
| ENSG00000260261 | AC124944.3 | 166.453404 | -1.55974563 | 0.30381349 | -5.13389202 | 0.0000 | 0.0002 |
| ENSG00000196735 | HLA-DQA1 | 887.782569 | -2.99971837 | 0.5852865 | -5.12521366 | 0.0000 | 0.0002 |
| ENSG00000267904 | AC024075.1 | 21.9153032 | 20.8011843 | 4.08210303 | 5.0957029 | 0.0000 | 0.0002 |
| ENSG00000104415 | WISP1 | 402.838858 | 2.73643291 | 0.53696182 | 5.0961406 | 0.0000 | 0.0002 |
| ENSG00000211677 | IGLC2 | 181.871224 | -4.28434704 | 0.84191033 | -5.08884009 | 0.0000 | 0.0002 |
| ENSG00000211448 | DIO2 | 1627.83453 | 4.07401996 | 0.80115923 | 5.08515637 | 0.0000 | 0.0002 |
| ENSG00000169495 | HTRA4 | 9278.04448 | 2.498972 | 0.49232642 | 5.07584381 | 0.0000 | 0.0002 |
| ENSG00000159399 | HK2 | 1620.72934 | 2.48975774 | 0.49384266 | 5.04160116 | 0.0000 | 0.0002 |
| ENSG00000143632 | ACTA1 | 107.982393 | 3.48473164 | 0.69176185 | 5.03747302 | 0.0000 | 0.0002 |
| ENSG00000137440 | FGFBP1 | 82.2012013 | 5.70336071 | 1.1351011 | 5.02453983 | 0.0000 | 0.0002 |
| ENSG00000273003 | ARL2-SNX15 | 26.3123054 | 20.445355 | 4.08220786 | 5.00840618 | 0.0000 | 0.0003 |
| ENSG00000235609 | AF127577.4 | 599.015919 | 2.63473583 | 0.52747557 | 4.99499122 | 0.0000 | 0.0003 |
| ENSG00000259753 | AC068234.1 | 87.4554554 | 20.364062 | 4.08214433 | 4.98856982 | 0.0000 | 0.0003 |
| ENSG00000107485 | GATA3 | 10084.3781 | 1.37826948 | 0.27770591 | 4.96305424 | 0.0000 | 0.0003 |
| ENSG00000279789 | AC120114.4 | 291.23462 | -1.30839131 | 0.26467185 | -4.94344717 | 0.0000 | 0.0003 |
| ENSG00000052344 | PRSS8 | 12528.907 | 1.50224761 | 0.30455937 | 4.93252797 | 0.0000 | 0.0004 |
| ENSG00000261326 | LINC01355 | 1031.34719 | -1.4379264 | 0.29171956 | -4.92913951 | 0.0000 | 0.0004 |
| ENSG00000267172 | AC022031.1 | 14.0611548 | -7.55168369 | 1.54111689 | -4.90013687 | 0.0000 | 0.0004 |
| ENSG00000280083 | AC079777.1 | 72.7272268 | -3.96572588 | 0.81168788 | -4.88577686 | 0.0000 | 0.0004 |
| ENSG00000075673 | ATP12A | 40.7956563 | -8.86655989 | 1.81591427 | -4.88269741 | 0.0000 | 0.0004 |
| ENSG00000116017 | ARID3A | 12516.8681 | 0.93853645 | 0.19277593 | 4.86853542 | 0.0000 | 0.0005 |
| ENSG00000130147 | SH3BP4 | 3328.66087 | 1.09321401 | 0.22471955 | 4.86479253 | 0.0000 | 0.0005 |
| ENSG00000227388 | AL133410.1 | 12.8019441 | -7.18590731 | 1.48244686 | -4.84732878 | 0.0000 | 0.0005 |
| ENSG00000170290 | SLN | 25.5434466 | 5.93846493 | 1.2235869 | 4.85332503 | 0.0000 | 0.0005 |

| | | | | | | | |
|---|---|---|---|---|---|---|---|
| ENSG00000185442 | FAM174B | 602.038221 | 1.73433461 | 0.35759663 | 4.84997475 | 0.0000 | 0.0005 |
| ENSG00000112902 | SEMA5A | 2125.82123 | 2.1932617 | 0.45172261 | 4.85532855 | 0.0000 | 0.0005 |
| ENSG00000105281 | SLC1A5 | 5439.19613 | 1.56043783 | 0.32167051 | 4.85104409 | 0.0000 | 0.0005 |
| ENSG00000246985 | SOCS2-AS1 | 425.693367 | -1.34178089 | 0.27704928 | -4.84311271 | 0.0000 | 0.0005 |
| ENSG00000134107 | BHLHE40 | 11675.2292 | 2.31014792 | 0.47685719 | 4.84452781 | 0.0000 | 0.0005 |
| ENSG00000163430 | FSTL1 | 59301.2976 | 1.29108331 | 0.26725059 | 4.83098396 | 0.0000 | 0.0005 |
| ENSG00000129038 | LOXL1 | 2279.67113 | 1.14442211 | 0.23705274 | 4.82771105 | 0.0000 | 0.0005 |
| ENSG00000132613 | MTSS1L | 11467.8171 | 2.01655856 | 0.42026443 | 4.79830892 | 0.0000 | 0.0005 |
| ENSG00000109814 | UGDH | 2484.4118 | 1.49087431 | 0.31067278 | 4.79885728 | 0.0000 | 0.0005 |
| ENSG00000234290 | AC116366.1 | 238.302305 | -1.49375584 | 0.31262286 | -4.77814009 | 0.0000 | 0.0006 |
| ENSG00000141526 | SLC16A3 | 10509.2182 | 1.30844596 | 0.27509814 | 4.75628785 | 0.0000 | 0.0007 |
| ENSG00000075275 | CELSR1 | 620.662087 | 2.84364277 | 0.60031195 | 4.73694178 | 0.0000 | 0.0007 |
| ENSG00000175315 | CST6 | 1294.53671 | 3.49344357 | 0.73822647 | 4.73221118 | 0.0000 | 0.0007 |
| ENSG00000139514 | SLC7A1 | 2633.16021 | 1.88722883 | 0.39968224 | 4.72182304 | 0.0000 | 0.0007 |
| ENSG00000166387 | PPFIBP2 | 1162.60093 | 1.12164035 | 0.23854441 | 4.70201905 | 0.0000 | 0.0008 |
| ENSG00000198848 | CES1 | 291.451184 | 1.98786172 | 0.4231614 | 4.69764426 | 0.0000 | 0.0008 |
| ENSG00000128203 | ASPHD2 | 277.457431 | 2.58055478 | 0.55022147 | 4.69002922 | 0.0000 | 0.0008 |
| ENSG00000147655 | RSPO2 | 395.688707 | 3.31379045 | 0.70677487 | 4.68860819 | 0.0000 | 0.0008 |
| ENSG00000269124 | AC007193.2 | 54.3423458 | -2.76945745 | 0.59210683 | -4.67729357 | 0.0000 | 0.0009 |
| ENSG00000153822 | KCNJ16 | 118.390697 | 2.95497792 | 0.63312215 | 4.66731087 | 0.0000 | 0.0009 |
| ENSG00000253939 | AC007991.3 | 64.9028815 | -2.77410053 | 0.59737194 | -4.64384141 | 0.0000 | 0.0010 |
| ENSG00000183691 | NOG | 4390.8474 | 3.38965326 | 0.73238189 | 4.62825922 | 0.0000 | 0.0011 |
| ENSG00000130635 | COL5A1 | 27082.1653 | 1.8990692 | 0.41080945 | 4.62274958 | 0.0000 | 0.0011 |
| ENSG00000124731 | TREM1 | 1024.66176 | 3.33590595 | 0.72530759 | 4.59929825 | 0.0000 | 0.0012 |
| ENSG00000121858 | TNFSF10 | 8880.8521 | 1.54763724 | 0.33670749 | 4.59638498 | 0.0000 | 0.0012 |
| ENSG00000099812 | MISP | 352.648747 | 3.75220829 | 0.81693333 | 4.593041 | 0.0000 | 0.0012 |
| ENSG00000174697 | LEP | 17214.7911 | 5.32675521 | 1.16258727 | 4.58181106 | 0.0000 | 0.0013 |
| ENSG00000271327 | AC010201.2 | 52.2334199 | -2.53687858 | 0.55413372 | -4.57809816 | 0.0000 | 0.0013 |
| ENSG00000243302 | AC018638.4 | 1058.10114 | -1.45697591 | 0.31853633 | -4.57397099 | 0.0000 | 0.0013 |
| ENSG00000197122 | SRC | 2206.01114 | 0.7201999 | 0.15763818 | 4.56868944 | 0.0000 | 0.0013 |
| ENSG00000226239 | AL031658.1 | 109.023588 | -2.22449479 | 0.48795121 | -4.55884672 | 0.0000 | 0.0014 |
| ENSG00000256276 | | 66.8588668 | 3.99278813 | 0.87765871 | 4.5493631 | 0.0000 | 0.0014 |
| ENSG00000211574 | MIR770 | 321.223042 | -2.4805347 | 0.54477664 | -4.55330592 | 0.0000 | 0.0014 |
| ENSG00000090776 | EFNB1 | 8244.78921 | 1.32004432 | 0.29004062 | 4.55123942 | 0.0000 | 0.0014 |
| ENSG00000198125 | MB | 18.420404 | 8.44820711 | 1.85988995 | 4.5423156 | 0.0000 | 0.0014 |
| ENSG00000161638 | ITGA5 | 31549.1764 | 1.39264003 | 0.30784681 | 4.52380854 | 0.0000 | 0.0015 |
| ENSG00000115339 | GALNT3 | 526.562996 | 2.02496676 | 0.44749288 | 4.52513735 | 0.0000 | 0.0015 |
| ENSG00000210077 | MT-TV | 166.282047 | 2.85611963 | 0.63086084 | 4.52733702 | 0.0000 | 0.0015 |
| ENSG00000129521 | EGLN3 | 2278.95575 | 3.14148507 | 0.69967574 | 4.48991564 | 0.0000 | 0.0017 |
| ENSG00000104154 | SLC30A4 | 703.064562 | 1.13285658 | 0.25266484 | 4.48363358 | 0.0000 | 0.0018 |
| ENSG00000128268 | MGAT3 | 3098.50076 | 1.58031519 | 0.3529829 | 4.4770305 | 0.0000 | 0.0018 |
| ENSG00000280213 | UCKL1-AS1 | 349.473789 | -1.4426283 | 0.32354612 | -4.45880263 | 0.0000 | 0.0019 |
| ENSG00000279759 | AC118344.2 | 330.026039 | -1.68662752 | 0.3782138 | -4.45945522 | 0.0000 | 0.0019 |
| ENSG00000113083 | LOX | 602.425102 | 2.4757672 | 0.55565121 | 4.45561378 | 0.0000 | 0.0019 |
| ENSG00000104419 | NDRG1 | 14313.1876 | 1.717585 | 0.38566111 | 4.45361219 | 0.0000 | 0.0019 |
| ENSG00000249321 | OR5H5P | 79.6224735 | 5.30738054 | 1.19353118 | 4.44678835 | 0.0000 | 0.0020 |
| ENSG00000223724 | RAD17P2 | 13.4568087 | -7.35536616 | 1.65505266 | -4.4441886 | 0.0000 | 0.0020 |
| ENSG00000231826 | LINC01819 | 132.830538 | 4.15441064 | 0.93571115 | 4.43984305 | 0.0000 | 0.0020 |
| ENSG00000102996 | MMP15 | 12126.9029 | 0.79066355 | 0.17807027 | 4.44017728 | 0.0000 | 0.0020 |
| ENSG00000136026 | CKAP4 | 5518.70177 | 0.94413506 | 0.21304063 | 4.43171367 | 0.0000 | 0.0020 |
| ENSG00000106366 | SERPINE1 | 87421.1606 | 2.18606221 | 0.49299163 | 4.43427854 | 0.0000 | 0.0020 |
| ENSG00000204381 | LAYN | 704.037477 | 1.44040177 | 0.32503767 | 4.43149174 | 0.0000 | 0.0020 |
| ENSG00000100234 | TIMP3 | 181582.935 | 2.15694435 | 0.48755967 | 4.42395972 | 0.0000 | 0.0021 |
| ENSG00000130822 | PNCK | 459.691469 | 2.48920572 | 0.56281327 | 4.42279142 | 0.0000 | 0.0021 |
| ENSG00000168938 | PPIC | 2410.44759 | 1.19628271 | 0.27118636 | 4.41129376 | 0.0000 | 0.0022 |
| ENSG00000049860 | HEXB | 24773.1517 | 1.46418798 | 0.33281536 | 4.39940026 | 0.0000 | 0.0023 |
| ENSG00000078140 | UBE2K | 2781.48234 | 1.78575786 | 0.40632378 | 4.39491348 | 0.0000 | 0.0023 |
| ENSG00000186847 | KRT14 | 1047.38984 | 4.92670143 | 1.12608012 | 4.37508961 | 0.0000 | 0.0025 |
| ENSG00000065618 | COL17A1 | 10655.4415 | 3.08271315 | 0.70650394 | 4.36333467 | 0.0000 | 0.0026 |
| ENSG00000167394 | ZNF668 | 560.741231 | 1.11295065 | 0.25617802 | 4.34444241 | 0.0000 | 0.0028 |
| ENSG00000279204 | AC134043.2 | 70.768301 | -1.8514952 | 0.4271349 | -4.33468492 | 0.0000 | 0.0029 |
| ENSG00000147852 | VLDLR | 1369.58423 | 1.00694856 | 0.23262085 | 4.32871158 | 0.0000 | 0.0030 |
| ENSG00000136156 | ITM2B | 69489.6773 | 1.47725544 | 0.3414789 | 4.32605193 | 0.0000 | 0.0030 |
| ENSG00000280163 | AC040160.2 | 48.7305495 | -2.48032724 | 0.57498913 | -4.3136941 | 0.0000 | 0.0031 |
| ENSG00000235162 | C12orf75 | 1531.31112 | 2.55565688 | 0.59256335 | 4.31288383 | 0.0000 | 0.0031 |
| ENSG00000229951 | AC104695.2 | 64.9932957 | 1.82820858 | 0.42467451 | 4.30496421 | 0.0000 | 0.0032 |
| ENSG00000161714 | PLCD3 | 2114.56037 | 1.65662436 | 0.38468497 | 4.30644421 | 0.0000 | 0.0032 |
| ENSG00000115138 | POMC | 229.8462 | 2.87461563 | 0.66971731 | 4.2922821 | 0.0000 | 0.0032 |
| ENSG00000229413 | AC018638.1 | 547.025205 | -1.67310608 | 0.3889078 | -4.30206357 | 0.0000 | 0.0032 |
| ENSG00000157227 | MMP14 | 24328.2879 | 0.95687277 | 0.22286447 | 4.29351871 | 0.0000 | 0.0032 |
| ENSG00000116260 | QSOX1 | 42702.2138 | 2.07450598 | 0.48281748 | 4.29666708 | 0.0000 | 0.0032 |

| | | | | | | | |
|---|---|---|---|---|---|---|---|
| ENSG00000127124 | HIVEP3 | 456.135114 | 1.42837195 | 0.33281087 | 4.29184284 | 0.0000 | 0.0032 |
| ENSG00000167136 | ENDOG | 580.127352 | 1.71972429 | 0.39989166 | 4.30047548 | 0.0000 | 0.0032 |
| ENSG00000012232 | EXTL3 | 4225.48146 | 1.81279118 | 0.42205226 | 4.29518184 | 0.0000 | 0.0032 |
| ENSG00000115963 | RND3 | 2558.75708 | 1.36644076 | 0.31824605 | 4.2936613 | 0.0000 | 0.0032 |
| ENSG00000224728 | IMPDH1P8 | 9.82723625 | -6.85916464 | 1.59895556 | -4.28977817 | 0.0000 | 0.0032 |
| ENSG00000272505 | AC104964.4 | 53.9615611 | -2.11519003 | 0.49374559 | -4.28396743 | 0.0000 | 0.0033 |
| ENSG00000242600 | MBL1P | 90.704403 | -2.29096611 | 0.53503442 | -4.28190419 | 0.0000 | 0.0033 |
| ENSG00000061656 | SPAG4 | 445.792854 | 1.87125555 | 0.43731385 | 4.27897614 | 0.0000 | 0.0033 |
| ENSG00000271430 | | 1516.49252 | -0.9020825 | 0.21087458 | -4.27781526 | 0.0000 | 0.0033 |
| ENSG00000280334 | AC009084.2 | 176.644414 | -2.21840627 | 0.51920871 | -4.27266766 | 0.0000 | 0.0033 |
| ENSG00000226871 | AC135178.1 | 13.7338091 | -6.68802524 | 1.56518127 | -4.27300362 | 0.0000 | 0.0033 |
| ENSG00000169862 | CTNND2 | 114.43094 | 2.65971285 | 0.6236388 | 4.26482905 | 0.0000 | 0.0034 |
| ENSG00000011028 | MRC2 | 3088.01444 | 1.97551129 | 0.46359662 | 4.26127201 | 0.0000 | 0.0034 |
| ENSG00000135074 | ADAM19 | 6578.80516 | 1.67362163 | 0.39462435 | 4.24105009 | 0.0000 | 0.0037 |
| ENSG00000135919 | SERPINE2 | 73020.2083 | 2.75018448 | 0.6495433 | 4.23402792 | 0.0000 | 0.0038 |
| ENSG00000189159 | JPT1 | 3748.10663 | 2.08619703 | 0.49274765 | 4.23380413 | 0.0000 | 0.0038 |
| ENSG00000211679 | IGLC3 | 108.941708 | -3.87143602 | 0.91605152 | -4.22622084 | 0.0000 | 0.0039 |
| ENSG00000142949 | PTPRF | 44447.5844 | 1.56984794 | 0.37149602 | 4.22574636 | 0.0000 | 0.0039 |
| ENSG00000188910 | GJB3 | 285.19332 | 3.10204737 | 0.73441909 | 4.2238109 | 0.0000 | 0.0039 |
| ENSG00000185269 | NOTUM | 44555.6252 | 2.76636157 | 0.65656784 | 4.21336746 | 0.0000 | 0.0041 |
| ENSG00000174705 | SH3PXD2B | 2662.29139 | 1.82212133 | 0.43271944 | 4.21086084 | 0.0000 | 0.0041 |
| ENSG00000105048 | TNNT1 | 138.115403 | 2.95836971 | 0.70369348 | 4.20406013 | 0.0000 | 0.0041 |
| ENSG00000172379 | ARNT2 | 685.482169 | 2.60955114 | 0.62032239 | 4.20676603 | 0.0000 | 0.0041 |
| ENSG00000281991 | TMEM265 | 94.6018094 | 1.91815081 | 0.45625072 | 4.2041595 | 0.0000 | 0.0041 |
| ENSG00000168539 | CHRM1 | 15.1928658 | -7.16218254 | 1.70772931 | -4.19397999 | 0.0000 | 0.0042 |
| ENSG00000280383 | Z95331.1 | 127.433968 | -1.59908964 | 0.38128881 | -4.1939065 | 0.0000 | 0.0042 |
| ENSG00000137809 | ITGA11 | 522.730825 | 2.43494134 | 0.58092051 | 4.19152245 | 0.0000 | 0.0042 |
| ENSG00000183734 | ASCL2 | 2031.50905 | 2.89712578 | 0.69044392 | 4.19603347 | 0.0000 | 0.0042 |
| ENSG00000225855 | RUSC1-AS1 | 423.509129 | -1.57891416 | 0.37673549 | -4.19104167 | 0.0000 | 0.0042 |
| ENSG00000139908 | TSSK4 | 83.3113195 | -2.53437271 | 0.60485836 | -4.19002673 | 0.0000 | 0.0042 |
| ENSG00000184999 | SLC22A10 | 11.9197455 | -6.88670508 | 1.63989736 | -4.19947325 | 0.0000 | 0.0042 |
| ENSG00000178150 | ZNF114 | 76.0089342 | 2.63505611 | 0.62878499 | 4.19071089 | 0.0000 | 0.0042 |
| ENSG00000174640 | SLCO2A1 | 11626.5352 | 1.51840261 | 0.3622613 | 4.19145684 | 0.0000 | 0.0042 |
| ENSG00000179344 | HLA-DQB1 | 1577.47823 | -3.07679144 | 0.73457618 | -4.18852599 | 0.0000 | 0.0042 |
| ENSG00000172458 | IL17D | 91.7651948 | 2.60579252 | 0.62240859 | 4.18662684 | 0.0000 | 0.0042 |
| ENSG00000174353 | STAG3L3 | 1692.40166 | -1.93124286 | 0.46151528 | -4.18456973 | 0.0000 | 0.0042 |
| ENSG00000266865 | AC138207.8 | 55.1120539 | -1.57020993 | 0.37662223 | -4.16919082 | 0.0000 | 0.0044 |
| ENSG00000279569 | AC020763.4 | 709.367724 | 2.1896995 | 0.52514693 | 4.16968926 | 0.0000 | 0.0044 |
| ENSG00000131370 | SH3BP5 | 5361.57146 | 1.46929245 | 0.35294046 | 4.16300376 | 0.0000 | 0.0045 |
| ENSG00000197565 | COL4A6 | 298.588762 | 1.81248762 | 0.43624849 | 4.15471379 | 0.0000 | 0.0047 |
| ENSG00000210049 | MT-TF | 139.274864 | 2.16061567 | 0.52066896 | 4.14969169 | 0.0000 | 0.0047 |
| ENSG00000102034 | ELF4 | 3931.55809 | 1.31325214 | 0.3164687 | 4.14970621 | 0.0000 | 0.0047 |
| ENSG00000105877 | DNAH11 | 410.701152 | 3.3794735 | 0.81429673 | 4.15017447 | 0.0000 | 0.0047 |
| ENSG00000214049 | UCA1 | 1788.75021 | 2.79483742 | 0.67413463 | 4.14581492 | 0.0000 | 0.0047 |
| ENSG00000119698 | PPP4R4 | 76.5651743 | 3.34665132 | 0.80710123 | 4.14650753 | 0.0000 | 0.0047 |
| ENSG00000197540 | GZMM | 48.0807002 | -2.081597 | 0.50227691 | -4.14432151 | 0.0000 | 0.0047 |
| ENSG00000248112 | AC108174.1 | 57.863207 | 3.63552547 | 0.87853077 | 4.138188 | 0.0000 | 0.0048 |
| ENSG00000186466 | AQP7P1 | 61.8123488 | -3.35515044 | 0.811268 | -4.13568688 | 0.0000 | 0.0049 |
| ENSG00000255398 | HCAR3 | 146.93511 | 2.79824035 | 0.6776186 | 4.12952114 | 0.0000 | 0.0050 |
| ENSG00000120833 | SOCS2 | 2084.57763 | -1.14004139 | 0.27647416 | -4.12350071 | 0.0000 | 0.0051 |
| ENSG00000272610 | MAGI1-IT1 | 12.9944302 | -5.44114273 | 1.32284425 | -4.11321495 | 0.0000 | 0.0052 |
| ENSG00000166949 | SMAD3 | 2805.88291 | 1.28327899 | 0.31197881 | 4.11335306 | 0.0000 | 0.0052 |
| ENSG00000272983 | AL117339.4 | 38.7481603 | -2.29674097 | 0.55930235 | -4.10643899 | 0.0000 | 0.0054 |
| ENSG00000160392 | C19orf47 | 815.467915 | 1.21658421 | 0.29637495 | 4.10488208 | 0.0000 | 0.0054 |
| ENSG00000278434 | AC023830.3 | 8.53346114 | -6.56094457 | 1.59954292 | -4.10176212 | 0.0000 | 0.0054 |
| ENSG00000100196 | KDELR3 | 772.823684 | 2.03286779 | 0.49659191 | 4.09363853 | 0.0000 | 0.0056 |
| ENSG00000226312 | CFLAR-AS1 | 94.7737256 | -1.73075978 | 0.42268006 | -4.09472777 | 0.0000 | 0.0056 |
| ENSG00000158825 | CDA | 1205.66257 | 1.9301237 | 0.47168408 | 4.09198397 | 0.0000 | 0.0056 |
| ENSG00000105509 | HAS1 | 75.8016737 | 3.96797011 | 0.97235015 | 4.0808037 | 0.0000 | 0.0058 |
| ENSG00000131435 | PDLIM4 | 1637.87795 | 1.49688282 | 0.36721065 | 4.07636009 | 0.0000 | 0.0059 |
| ENSG00000215193 | PEX26 | 1543.9064 | -0.86339007 | 0.21174547 | -4.07749009 | 0.0000 | 0.0059 |
| ENSG00000008441 | NFIX | 1133.45251 | 1.70117108 | 0.41844947 | 4.0654158 | 0.0000 | 0.0061 |
| ENSG00000078269 | SYNJ2 | 1556.66808 | 1.02899697 | 0.25345678 | 4.0598519 | 0.0000 | 0.0062 |
| ENSG00000049323 | LTBP1 | 14140.8274 | 1.41858032 | 0.34947845 | 4.05913536 | 0.0000 | 0.0062 |
| ENSG00000279019 | AC009090.4 | 96.1986037 | -2.69894417 | 0.66442449 | -4.06207811 | 0.0000 | 0.0062 |
| ENSG00000169429 | CXCL8 | 522.417183 | 2.85169745 | 0.70230152 | 4.06050301 | 0.0000 | 0.0062 |
| ENSG00000169891 | REPS2 | 531.04958 | 2.70232431 | 0.66658235 | 4.05399918 | 0.0001 | 0.0063 |
| ENSG00000130821 | SLC6A8 | 5018.45702 | 1.38147688 | 0.34134185 | 4.04719462 | 0.0001 | 0.0064 |
| ENSG00000231607 | DLEU2 | 301.266025 | -1.75195267 | 0.43351488 | -4.0412746 | 0.0001 | 0.0065 |
| ENSG00000134352 | IL6ST | 26705.3695 | 1.36659769 | 0.33820892 | 4.0406908 | 0.0001 | 0.0065 |
| ENSG00000196268 | ZNF493 | 203.014933 | -1.49784726 | 0.37117622 | -4.0354074 | 0.0001 | 0.0066 |

| | | | | | | | |
|---|---|---|---|---|---|---|---|
| ENSG00000212907 | MT-ND4L | 100847.558 | 1.78451089 | 0.44223801 | 4.03518204 | 0.0001 | 0.0066 |
| ENSG00000228513 | AC023271.1 | 9.7417613 | -6.85543425 | 1.70045608 | -4.03152679 | 0.0001 | 0.0067 |
| ENSG00000208005 | MIR503 | 304.754336 | -1.79828131 | 0.44660333 | -4.02657387 | 0.0001 | 0.0068 |
| ENSG00000148498 | PARD3 | 2142.93047 | 0.91962633 | 0.22853756 | 4.02396138 | 0.0001 | 0.0068 |
| ENSG00000275216 | AL161431.1 | 817.477498 | 2.18320743 | 0.54253541 | 4.02408287 | 0.0001 | 0.0068 |
| ENSG00000263272 | AC004148.2 | 478.309598 | -1.02789822 | 0.25573527 | -4.01938386 | 0.0001 | 0.0069 |
| ENSG00000177791 | MYOZ1 | 186.339233 | 3.1062593 | 0.77380769 | 4.01425228 | 0.0001 | 0.0070 |
| ENSG00000147872 | PLIN2 | 19077.7365 | 2.35040919 | 0.58584152 | 4.01202221 | 0.0001 | 0.0071 |
| ENSG00000002726 | AOC1 | 115851.308 | 2.36025227 | 0.58903268 | 4.00699715 | 0.0001 | 0.0072 |
| ENSG00000183010 | PYCR1 | 1037.69672 | 2.01924536 | 0.50423676 | 4.00455801 | 0.0001 | 0.0072 |
| ENSG00000119715 | ESRRB | 42.8279705 | -2.63400587 | 0.65858349 | -3.99950179 | 0.0001 | 0.0074 |
| ENSG00000259645 | AC027237.4 | 6.575484 | -6.34089281 | 1.58763076 | -3.99393422 | 0.0001 | 0.0075 |
| ENSG00000127377 | CRYGN | 72.0074954 | 2.59410643 | 0.65245011 | 3.97594603 | 0.0001 | 0.0080 |
| ENSG00000115414 | FN1 | 732485.962 | 2.7000525 | 0.67923986 | 3.97510901 | 0.0001 | 0.0080 |
| ENSG00000013588 | GPRC5A | 2374.42706 | 2.82105278 | 0.7100062 | 3.97327906 | 0.0001 | 0.0081 |
| ENSG00000137267 | TUBB2A | 2335.68845 | 2.20215123 | 0.55473145 | 3.96976093 | 0.0001 | 0.0081 |
| ENSG00000122042 | UBL3 | 3251.83055 | 1.39259326 | 0.35135836 | 3.96345565 | 0.0001 | 0.0082 |
| ENSG00000101856 | PGRMC1 | 3109.63067 | 1.01629829 | 0.25635004 | 3.96449439 | 0.0001 | 0.0082 |
| ENSG00000188153 | COL4A5 | 1340.73029 | 0.81360454 | 0.20512696 | 3.96634625 | 0.0001 | 0.0082 |
| ENSG00000232611 | AL683813.1 | 112.315748 | -1.59068938 | 0.40127131 | -3.96412438 | 0.0001 | 0.0082 |
| ENSG00000146966 | DENND2A | 1683.13394 | 1.49387562 | 0.37779111 | 3.95423706 | 0.0001 | 0.0085 |
| ENSG00000224057 | EGFR-AS1 | 775.713218 | 2.54989101 | 0.64646598 | 3.9443545 | 0.0001 | 0.0088 |
| ENSG00000139988 | RDH12 | 19.5914343 | -5.08740887 | 1.29175462 | -3.93837096 | 0.0001 | 0.0090 |
| ENSG00000283236 | AC074141.1 | 70.4275466 | -1.97757022 | 0.50240751 | -3.93618763 | 0.0001 | 0.0090 |
| ENSG00000143333 | RGS16 | 645.052163 | 1.95888275 | 0.49939191 | 3.92253598 | 0.0001 | 0.0094 |
| ENSG00000279162 | AC141586.5 | 62.6315671 | -1.67922651 | 0.42785149 | -3.92478825 | 0.0001 | 0.0094 |
| ENSG00000189253 | TRIM64B | 233.722362 | 5.92250883 | 1.50978143 | 3.9227591 | 0.0001 | 0.0094 |
| ENSG00000070614 | NDST1 | 4520.14186 | 0.91090552 | 0.23220192 | 3.92290269 | 0.0001 | 0.0094 |
| ENSG00000186907 | RTN4RL2 | 356.150731 | 2.59892636 | 0.66389057 | 3.91469087 | 0.0001 | 0.0096 |
| ENSG00000197182 | MIRLET7BHG | 2043.85522 | -1.44494212 | 0.36905766 | -3.91522054 | 0.0001 | 0.0096 |
| ENSG00000136859 | ANGPTL2 | 979.323331 | 1.46530913 | 0.37540393 | 3.90328661 | 0.0001 | 0.0101 |
| ENSG00000128342 | LIF | 706.630512 | 4.3402906 | 1.11444969 | 3.89455946 | 0.0001 | 0.0104 |
| ENSG00000149346 | SLX4IP | 437.019152 | -0.9041942 | 0.23234643 | -3.89157777 | 0.0001 | 0.0105 |
| ENSG00000124225 | PMEPA1 | 1004.56522 | 1.93926571 | 0.4983962 | 3.89101224 | 0.0001 | 0.0105 |
| ENSG00000228695 | CES1P1 | 46.5524903 | 2.36430707 | 0.60789464 | 3.88933694 | 0.0001 | 0.0105 |
| ENSG00000074590 | NUAK1 | 955.11285 | 1.80434287 | 0.46437193 | 3.88555539 | 0.0001 | 0.0106 |
| ENSG00000164176 | EDIL3 | 443.69517 | 2.01515115 | 0.51875037 | 3.88462593 | 0.0001 | 0.0106 |
| ENSG00000243679 | AC018638.5 | 1852.78593 | -1.30968805 | 0.33717826 | -3.88426 | 0.0001 | 0.0106 |
| ENSG00000188227 | ZNF793 | 261.574274 | -2.58975552 | 0.66711868 | -3.88200121 | 0.0001 | 0.0106 |
| ENSG00000164849 | GPR146 | 1111.37252 | 1.4556834 | 0.37502773 | 3.88153539 | 0.0001 | 0.0106 |
| ENSG00000254818 | AP004607.3 | 11.3373896 | 7.82561647 | 2.01935212 | 3.87531049 | 0.0001 | 0.0107 |
| ENSG00000257151 | PWAR6 | 106.685268 | -1.6457401 | 0.42492121 | -3.87304767 | 0.0001 | 0.0107 |
| ENSG00000258818 | RNASE4 | 267.578032 | 2.0264371 | 0.52324539 | 3.87282364 | 0.0001 | 0.0107 |
| ENSG00000210112 | MT-TM | 245.692412 | 2.35854535 | 0.60848665 | 3.87608393 | 0.0001 | 0.0107 |
| ENSG00000176532 | PRR15 | 648.882019 | 2.1476341 | 0.55405407 | 3.8762175 | 0.0001 | 0.0107 |
| ENSG00000186951 | PPARA | 626.872599 | 1.41328409 | 0.36490531 | 3.87301597 | 0.0001 | 0.0107 |
| ENSG00000108821 | COL1A1 | 117644.829 | 1.79675511 | 0.46359062 | 3.87573659 | 0.0001 | 0.0107 |
| ENSG00000225345 | SNX18P3 | 12.6443222 | 5.45891977 | 1.40790089 | 3.87734663 | 0.0001 | 0.0107 |
| ENSG00000116574 | RHOU | 5878.68533 | 1.12873563 | 0.29155143 | 3.87148036 | 0.0001 | 0.0107 |
| ENSG00000146070 | PLA2G7 | 807.480711 | 2.66517722 | 0.688705 | 3.86983862 | 0.0001 | 0.0107 |
| ENSG00000165030 | NFIL3 | 3182.55966 | 1.3758767 | 0.35644941 | 3.85994938 | 0.0001 | 0.0111 |
| ENSG00000231468 | PRDX3P2 | 8.61944166 | -6.23677478 | 1.61765529 | -3.85544116 | 0.0001 | 0.0113 |
| ENSG00000156515 | HK1 | 6978.43263 | 0.69583399 | 0.18086506 | 3.84725486 | 0.0001 | 0.0116 |
| ENSG00000206557 | TRIM71 | 168.071283 | 2.49085558 | 0.64779921 | 3.84510435 | 0.0001 | 0.0117 |
| ENSG00000136720 | HS6ST1 | 3623.10722 | 1.34730213 | 0.35050935 | 3.84384074 | 0.0001 | 0.0117 |
| ENSG00000074416 | MGLL | 7199.72329 | 1.05802585 | 0.27536297 | 3.84229536 | 0.0001 | 0.0117 |
| ENSG00000243701 | DUBR | 1022.55645 | -0.84462137 | 0.22016198 | -3.83636346 | 0.0001 | 0.0119 |
| ENSG00000166592 | RRAD | 4646.31295 | 1.99206367 | 0.51965371 | 3.83344456 | 0.0001 | 0.0119 |
| ENSG00000104848 | KCNA7 | 72.2397038 | -4.43717711 | 1.15727082 | -3.83417349 | 0.0001 | 0.0119 |
| ENSG00000278784 | AL136295.7 | 262.730391 | -1.16274441 | 0.30320679 | -3.83482313 | 0.0001 | 0.0119 |
| ENSG00000155254 | MARVELD1 | 4065.56832 | 0.89128146 | 0.23267061 | 3.83065763 | 0.0001 | 0.0120 |
| ENSG00000259838 | ELOCP2 | 22.5818833 | -7.00910684 | 1.83095456 | -3.82811621 | 0.0001 | 0.0121 |
| ENSG00000263327 | TAPT1-AS1 | 155.547524 | -2.0420642 | 0.53393488 | -3.82455664 | 0.0001 | 0.0123 |
| ENSG00000072682 | P4HA2 | 2015.59216 | 1.2929753 | 0.33840368 | 3.82080749 | 0.0001 | 0.0124 |
| ENSG00000157766 | ACAN | 160.872239 | 1.97530803 | 0.51717605 | 3.81941123 | 0.0001 | 0.0124 |
| ENSG00000206417 | H1FX-AS1 | 300.607973 | -1.27841246 | 0.33490157 | -3.81727825 | 0.0001 | 0.0124 |
| ENSG00000175793 | SFN | 394.985239 | 3.41953778 | 0.89566932 | 3.81785745 | 0.0001 | 0.0124 |
| ENSG00000242735 | RPSAP26 | 92.5530685 | -1.6588736 | 0.43484186 | -3.81488942 | 0.0001 | 0.0125 |
| ENSG00000224086 | AC245452.1 | 641.87247 | -1.01188727 | 0.26542272 | -3.81236123 | 0.0001 | 0.0126 |
| ENSG00000233559 | LINC00513 | 23.9101727 | -3.33257194 | 0.87484672 | -3.80932095 | 0.0001 | 0.0127 |
| ENSG00000183535 | COL18A1-AS1 | 15.7535564 | -7.59300483 | 1.99501233 | -3.80599393 | 0.0001 | 0.0128 |

| | | | | | | | |
|---|---|---|---|---|---|---|---|
| ENSG00000058085 | LAMC2 | 2430.41576 | 1.73959854 | 0.45721289 | 3.80478891 | 0.0001 | 0.0128 |
| ENSG00000210156 | MT-TK | 173.128585 | 1.80446765 | 0.47453477 | 3.80260363 | 0.0001 | 0.0129 |
| ENSG00000234444 | ZNF736 | 549.875686 | -2.70439855 | 0.71138926 | -3.80157349 | 0.0001 | 0.0129 |
| ENSG00000084072 | PPIE | 1826.77909 | -1.05139916 | 0.27675475 | -3.79902848 | 0.0001 | 0.0130 |
| ENSG00000179477 | ALOX12B | 6.39516401 | -6.31747513 | 1.66329494 | -3.7981689 | 0.0001 | 0.0130 |
| ENSG00000243004 | AC005062.1 | 824.910533 | 1.22623489 | 0.32398305 | 3.78487365 | 0.0002 | 0.0136 |
| ENSG00000206120 | EGFEM1P | 67.7349553 | -1.84282402 | 0.48678115 | -3.78573418 | 0.0002 | 0.0136 |
| ENSG00000174403 | MIR1-1HG-AS1 | 16.9274601 | 7.30870205 | 1.9331117 | 3.78079655 | 0.0002 | 0.0138 |
| ENSG00000134265 | NAPG | 1336.40539 | -1.13025958 | 0.29901238 | -3.77997581 | 0.0002 | 0.0138 |
| ENSG00000187231 | SESTD1 | 1733.74297 | 1.18047823 | 0.31248842 | 3.77767033 | 0.0002 | 0.0138 |
| ENSG00000167634 | NLRP7 | 52.3616849 | 3.49801395 | 0.92618981 | 3.77677869 | 0.0002 | 0.0138 |
| ENSG00000211592 | IGKC | 542.753034 | -4.18850101 | 1.10876977 | -3.77761111 | 0.0002 | 0.0138 |
| ENSG00000140479 | PCSK6 | 3436.04594 | 2.00154208 | 0.53049268 | 3.77298716 | 0.0002 | 0.0140 |
| ENSG00000183230 | CTNNA3 | 9.81534659 | 6.43524672 | 1.70584758 | 3.7724629 | 0.0002 | 0.0140 |
| ENSG00000260266 | PPIAP46 | 1586.88396 | 2.09086669 | 0.55444984 | 3.77106552 | 0.0002 | 0.0140 |
| ENSG00000126215 | XRCC3 | 1164.49918 | -0.76452657 | 0.20295909 | -3.76689979 | 0.0002 | 0.0142 |
| ENSG00000177283 | FZD8 | 251.216782 | 1.51679443 | 0.40274116 | 3.76617689 | 0.0002 | 0.0142 |
| ENSG00000139629 | GALNT6 | 2149.35688 | 1.86058805 | 0.49481981 | 3.76013251 | 0.0002 | 0.0145 |
| ENSG00000158055 | GRHL3 | 172.895531 | 1.31430145 | 0.34957373 | 3.75972607 | 0.0002 | 0.0145 |
| ENSG00000152931 | PART1 | 517.270811 | -1.31797336 | 0.35067349 | -3.75840598 | 0.0002 | 0.0145 |
| ENSG00000173464 | RNASE11 | 15.3911274 | -7.76435954 | 2.06633896 | -3.75754399 | 0.0002 | 0.0145 |
| ENSG00000273329 | AC078846.1 | 250.775459 | -1.487916 | 0.39633502 | -3.75418752 | 0.0002 | 0.0146 |
| ENSG00000270661 | Z99289.2 | 9.03810508 | -6.8098509 | 1.81409063 | -3.75386476 | 0.0002 | 0.0146 |
| ENSG00000227051 | C14orf132 | 177.957314 | 1.68315061 | 0.44858571 | 3.75212711 | 0.0002 | 0.0147 |
| ENSG00000068781 | STON1-GTF2A1L | 144.578581 | 3.24809628 | 0.8670102 | 3.74631841 | 0.0002 | 0.0150 |
| ENSG00000231925 | TAPBP | 9913.6306 | 0.87741387 | 0.23474021 | 3.73780809 | 0.0002 | 0.0154 |
| ENSG00000172901 | LVRN | 14789.4838 | 2.51084826 | 0.67262988 | 3.73288242 | 0.0002 | 0.0157 |
| ENSG00000173801 | JUP | 15015.6973 | 0.98672666 | 0.26457039 | 3.72954302 | 0.0002 | 0.0158 |
| ENSG00000084731 | KIF3C | 443.218772 | 1.75378435 | 0.47050539 | 3.72744796 | 0.0002 | 0.0159 |
| ENSG00000184956 | MUC6 | 26.5668761 | -3.70711305 | 0.99481503 | -3.72643451 | 0.0002 | 0.0159 |
| ENSG00000112977 | DAP | 5278.07533 | 0.94151773 | 0.25265156 | 3.72654623 | 0.0002 | 0.0159 |
| ENSG00000173702 | MUC13 | 8.3879438 | -6.51921463 | 1.74986765 | -3.72554726 | 0.0002 | 0.0159 |
| ENSG00000103489 | XYLT1 | 1973.63156 | 1.35135638 | 0.36298871 | 3.72286064 | 0.0002 | 0.0160 |
| ENSG00000230715 | AC018638.2 | 618.568086 | -1.22971575 | 0.33050907 | -3.72067178 | 0.0002 | 0.0161 |
| ENSG00000171446 | KRT27 | 16.2820638 | -6.81890148 | 1.83414408 | -3.71775671 | 0.0002 | 0.0162 |
| ENSG00000264456 | AC138207.4 | 180.1604 | -1.33133531 | 0.35823607 | -3.71636313 | 0.0002 | 0.0163 |
| ENSG00000106397 | PLOD3 | 6670.83663 | 0.89744506 | 0.24157238 | 3.71501516 | 0.0002 | 0.0163 |
| ENSG00000268621 | IGFL2-AS1 | 150.825951 | 3.02862264 | 0.81573285 | 3.71276287 | 0.0002 | 0.0164 |
| ENSG00000107957 | SH3PXD2A | 14394.5334 | 1.52741433 | 0.41172193 | 3.70982023 | 0.0002 | 0.0165 |
| ENSG00000229180 | AC006001.3 | 1275.16203 | -1.10694123 | 0.29837779 | -3.70986467 | 0.0002 | 0.0165 |
| ENSG00000265107 | GJA5 | 9898.90725 | 1.21864507 | 0.32902553 | 3.70380096 | 0.0002 | 0.0166 |
| ENSG00000113369 | ARRDC3 | 11144.1179 | 1.6125404 | 0.43533947 | 3.7040988 | 0.0002 | 0.0166 |
| ENSG00000224220 | AC104699.1 | 12.3317778 | -5.25797019 | 1.41998407 | -3.70283744 | 0.0002 | 0.0166 |
| ENSG00000004660 | CAMKK1 | 333.987784 | 1.04243011 | 0.281296 | 3.70581205 | 0.0002 | 0.0166 |
| ENSG00000148344 | PTGES | 6624.63676 | 1.45252738 | 0.39231083 | 3.70249117 | 0.0002 | 0.0166 |
| ENSG00000174945 | AMZ1 | 244.528004 | 2.49596247 | 0.67398741 | 3.7032776 | 0.0002 | 0.0166 |
| ENSG00000016082 | ISL1 | 642.351538 | -1.16820269 | 0.31540677 | -3.70379712 | 0.0002 | 0.0166 |
| ENSG00000050426 | LETMD1 | 1701.42262 | -0.68859926 | 0.18658667 | -3.69050624 | 0.0002 | 0.0173 |
| ENSG00000134333 | LDHA | 10150.7831 | 1.51840906 | 0.4115695 | 3.68931383 | 0.0002 | 0.0174 |
| ENSG00000168542 | COL3A1 | 125609.741 | 1.4871019 | 0.40370491 | 3.68363587 | 0.0002 | 0.0177 |
| ENSG00000154127 | UBASH3B | 3520.65345 | 1.42281337 | 0.38649463 | 3.68132765 | 0.0002 | 0.0178 |
| ENSG00000247095 | MIR210HG | 444.507077 | 2.9262212 | 0.79626258 | 3.67494501 | 0.0002 | 0.0181 |
| ENSG00000265136 | AC124283.4 | 8.04851209 | -5.95451873 | 1.62026189 | -3.67503473 | 0.0002 | 0.0181 |
| ENSG00000187243 | MAGED4B | 501.006923 | 1.7341744 | 0.47207619 | 3.67350535 | 0.0002 | 0.0182 |
| ENSG00000205740 | AL359878.1 | 137.904118 | -1.24886948 | 0.34021251 | -3.67085113 | 0.0002 | 0.0183 |
| ENSG00000168490 | PHYHIP | 717.866255 | 1.36462716 | 0.3718649 | 3.66968532 | 0.0002 | 0.0184 |
| ENSG00000182782 | HCAR2 | 357.688569 | 2.33519868 | 0.63659641 | 3.66825612 | 0.0002 | 0.0184 |
| ENSG00000167992 | VWCE | 1021.96897 | 1.98705912 | 0.54237451 | 3.66362923 | 0.0002 | 0.0187 |
| ENSG00000274995 | AC013564.1 | 6.20861404 | -6.55811794 | 1.79042071 | -3.66289214 | 0.0002 | 0.0187 |
| ENSG00000186081 | KRT5 | 53.045365 | 4.93326675 | 1.34744341 | 3.66120513 | 0.0003 | 0.0187 |
| ENSG00000197124 | ZNF682 | 216.507608 | -1.54357501 | 0.42177575 | -3.65970546 | 0.0003 | 0.0187 |
| ENSG00000183690 | EFHC2 | 60.4341373 | 3.04120805 | 0.83102614 | 3.65958168 | 0.0003 | 0.0187 |
| ENSG00000203706 | SERTAD4-AS1 | 135.226174 | 1.59379641 | 0.43552551 | 3.6594789 | 0.0003 | 0.0187 |
| ENSG00000123999 | INHA | 3060.4934 | 1.71347935 | 0.46851671 | 3.65724275 | 0.0003 | 0.0188 |
| ENSG00000088280 | ASAP3 | 3584.56197 | 1.79550565 | 0.49133912 | 3.65431037 | 0.0003 | 0.0190 |
| ENSG00000229591 | AC006017.1 | 24.6338972 | -3.12372474 | 0.85545063 | -3.65155466 | 0.0003 | 0.0191 |
| ENSG00000134318 | ROCK2 | 3731.15217 | 0.92399827 | 0.25336175 | 3.64695244 | 0.0003 | 0.0193 |
| ENSG00000108679 | LGALS3BP | 14892.2939 | 1.3669268 | 0.37483598 | 3.64673322 | 0.0003 | 0.0193 |
| ENSG00000101000 | PROCR | 4911.62516 | 1.57565659 | 0.431951 | 3.64776694 | 0.0003 | 0.0193 |
| ENSG00000272472 | AL512283.1 | 87.610428 | -3.28237382 | 0.90029502 | -3.64588692 | 0.0003 | 0.0193 |
| ENSG00000196526 | AFAP1 | 3318.74985 | 1.61839757 | 0.44409498 | 3.64425998 | 0.0003 | 0.0194 |

| | | | | | | | |
|---|---|---|---|---|---|---|---|
| ENSG00000188706 | ZDHHC9 | 1775.71293 | 1.20895327 | 0.33216525 | 3.63961389 | 0.0003 | 0.0196 |
| ENSG00000211949 | IGHV3-23 | 32.2123698 | -6.49731391 | 1.78502372 | -3.6399034 | 0.0003 | 0.0196 |
| ENSG00000209082 | MT-TL1 | 1065.24447 | 2.21214667 | 0.60846375 | 3.63562605 | 0.0003 | 0.0197 |
| ENSG00000156804 | FBXO32 | 2203.05868 | 1.29706942 | 0.35665986 | 3.63671263 | 0.0003 | 0.0197 |
| ENSG00000152127 | MGAT5 | 6661.49112 | 2.01909579 | 0.55503436 | 3.63778525 | 0.0003 | 0.0197 |
| ENSG00000156466 | GDF6 | 580.832031 | 1.5575589 | 0.4282296 | 3.63720516 | 0.0003 | 0.0197 |
| ENSG00000162929 | KIAA1841 | 357.212671 | -1.1827291 | 0.32529981 | -3.63581249 | 0.0003 | 0.0197 |
| ENSG00000187642 | PERM1 | 40.7206293 | 3.06081756 | 0.84260644 | 3.63255893 | 0.0003 | 0.0197 |
| ENSG00000141985 | SH3GL1 | 7761.55903 | 0.72108661 | 0.19845908 | 3.6334271 | 0.0003 | 0.0197 |
| ENSG00000262663 | AC087222.1 | 41.5127008 | -2.31337707 | 0.63672879 | -3.63322201 | 0.0003 | 0.0197 |
| ENSG00000143858 | SYT2 | 74.6570023 | -1.63398432 | 0.4500486 | -3.63068412 | 0.0003 | 0.0198 |
| ENSG00000120708 | TGFBI | 17528.0995 | 1.66098539 | 0.45778711 | 3.62829217 | 0.0003 | 0.0200 |
| ENSG00000167123 | CERCAM | 11893.8913 | 1.60975793 | 0.44398206 | 3.62572742 | 0.0003 | 0.0201 |
| ENSG00000182931 | WFDC10B | 11.5204487 | 6.77346219 | 1.86921092 | 3.62370139 | 0.0003 | 0.0202 |
| ENSG00000254558 | AP005435.1 | 17.6649312 | 5.41440848 | 1.49461525 | 3.62261021 | 0.0003 | 0.0202 |
| ENSG00000135002 | RFK | 2312.1501 | 1.2841013 | 0.3545143 | 3.62214244 | 0.0003 | 0.0202 |
| ENSG00000179520 | SLC17A8 | 82.9953204 | 4.13708618 | 1.14223159 | 3.62193292 | 0.0003 | 0.0202 |
| ENSG00000215417 | MIR17HG | 140.824471 | -2.10740778 | 0.58198528 | -3.62106716 | 0.0003 | 0.0202 |
| ENSG00000162241 | SLC25A45 | 796.951308 | -0.96218014 | 0.26594712 | -3.61793779 | 0.0003 | 0.0204 |
| ENSG00000144161 | ZC3H8 | 400.123404 | -0.89852494 | 0.24838999 | -3.61739594 | 0.0003 | 0.0204 |
| ENSG00000116132 | PRRX1 | 206.930808 | 2.32599411 | 0.64334646 | 3.61546111 | 0.0003 | 0.0205 |
| ENSG00000105143 | SLC1A6 | 145.944419 | 5.08932776 | 1.40826915 | 3.61388855 | 0.0003 | 0.0205 |
| ENSG00000067082 | KLF6 | 7990.56903 | 1.49953304 | 0.41512647 | 3.61223182 | 0.0003 | 0.0205 |
| ENSG00000228492 | RAB11FIP1P1 | 268.995386 | -1.32130186 | 0.36574916 | -3.61259032 | 0.0003 | 0.0205 |
| ENSG00000197245 | FAM110D | 183.60649 | -1.57824204 | 0.43705347 | -3.61109603 | 0.0003 | 0.0206 |
| ENSG00000087470 | DNM1L | 2259.67596 | 0.87152247 | 0.24140021 | 3.61028052 | 0.0003 | 0.0206 |
| ENSG00000243836 | WDR86-AS1 | 796.589764 | 2.13499426 | 0.59172059 | 3.60811216 | 0.0003 | 0.0207 |
| ENSG00000188522 | FAM83G | 403.327301 | 2.00865011 | 0.55667337 | 3.60831002 | 0.0003 | 0.0207 |
| ENSG00000076716 | GPC4 | 3983.42827 | 1.43554445 | 0.39817486 | 3.60531161 | 0.0003 | 0.0208 |
| ENSG00000169245 | CXCL10 | 376.753762 | -2.88822806 | 0.80193985 | -3.60155197 | 0.0003 | 0.0209 |
| ENSG00000179406 | LINC00174 | 556.353747 | -1.26501093 | 0.35116139 | -3.60236336 | 0.0003 | 0.0209 |
| ENSG00000029993 | HMGB3 | 20200.1501 | 1.12722219 | 0.31295454 | 3.60187197 | 0.0003 | 0.0209 |
| ENSG00000173578 | XCR1 | 548.36863 | 2.42606738 | 0.67353304 | 3.60200204 | 0.0003 | 0.0209 |
| ENSG00000268182 | SMIM17 | 9.97716226 | -7.30528101 | 2.03044721 | -3.59786799 | 0.0003 | 0.0211 |
| ENSG00000198886 | MT-ND4 | 513087.615 | 1.40300844 | 0.38992603 | 3.59813995 | 0.0003 | 0.0211 |
| ENSG00000215481 | BCRP3 | 22.6360548 | 4.12824779 | 1.14871614 | 3.59379281 | 0.0003 | 0.0214 |
| ENSG00000260990 | Z94057.1 | 8.00816121 | -5.93759722 | 1.65257102 | -3.59294527 | 0.0003 | 0.0214 |
| ENSG00000139055 | ERP27 | 127.275279 | 2.64222092 | 0.7355388 | 3.59222506 | 0.0003 | 0.0214 |
| ENSG00000198796 | ALPK2 | 128.294308 | 1.95049695 | 0.54359951 | 3.58811387 | 0.0003 | 0.0216 |
| ENSG00000146674 | IGFBP3 | 108177.418 | 1.62363698 | 0.45238906 | 3.58902799 | 0.0003 | 0.0216 |
| ENSG00000183723 | CMTM4 | 1493.19273 | 1.50334995 | 0.41893324 | 3.58851914 | 0.0003 | 0.0216 |
| ENSG00000196154 | S100A4 | 3121.02934 | 1.57373166 | 0.43988432 | 3.57760342 | 0.0003 | 0.0224 |
| ENSG00000138795 | LEF1 | 345.578803 | -1.40430883 | 0.39275827 | -3.57550418 | 0.0003 | 0.0225 |
| ENSG00000151090 | THRB | 687.190364 | 1.40526308 | 0.3931926 | 3.57398152 | 0.0004 | 0.0226 |
| ENSG00000129991 | TNNI3 | 11.9564729 | 6.82737969 | 1.91054044 | 3.57353321 | 0.0004 | 0.0226 |
| ENSG00000172081 | MOB3A | 3225.70913 | 0.82520801 | 0.23127072 | 3.56814741 | 0.0004 | 0.0230 |
| ENSG00000176134 | AL445665.1 | 17.1518727 | -3.86303547 | 1.08350036 | -3.56532919 | 0.0004 | 0.0231 |
| ENSG00000249906 | AC006487.1 | 23.1125666 | -4.3213382 | 1.2120492 | -3.56531582 | 0.0004 | 0.0231 |
| ENSG00000173898 | SPTBN2 | 572.442102 | 1.80579033 | 0.5066063 | 3.56448458 | 0.0004 | 0.0231 |
| ENSG00000277957 | SENP3-EIF4A1 | 650.801032 | -5.77401007 | 1.62026411 | -3.56362275 | 0.0004 | 0.0231 |
| ENSG00000254165 | AC090739.1 | 26.6593084 | -2.28685808 | 0.64231283 | -3.56034935 | 0.0004 | 0.0234 |
| ENSG00000236308 | AL138921.2 | 14.2914734 | -3.98144018 | 1.11864156 | -3.55917419 | 0.0004 | 0.0234 |
| ENSG00000042980 | ADAM28 | 313.457958 | 2.2280383 | 0.62617671 | 3.55816221 | 0.0004 | 0.0235 |
| ENSG00000154545 | MAGED4 | 481.957267 | 1.7234034 | 0.48463687 | 3.55607156 | 0.0004 | 0.0236 |
| ENSG00000213906 | LTB4R2 | 358.675082 | -1.63685476 | 0.46037342 | -3.55549367 | 0.0004 | 0.0236 |
| ENSG00000128422 | KRT17 | 896.79168 | 2.69712153 | 0.75933722 | 3.55194169 | 0.0004 | 0.0236 |
| ENSG00000128928 | IVD | 2313.21466 | -0.85777188 | 0.24136646 | -3.55381559 | 0.0004 | 0.0236 |
| ENSG00000006453 | BAIAP2L1 | 2432.71242 | 1.10111449 | 0.30978907 | 3.55440067 | 0.0004 | 0.0236 |
| ENSG00000180448 | ARHGAP45 | 9211.30867 | 2.07253674 | 0.58340387 | 3.55249058 | 0.0004 | 0.0236 |
| ENSG00000245970 | AP003352.1 | 394.316535 | -0.90153837 | 0.25373812 | -3.55302689 | 0.0004 | 0.0236 |
| ENSG00000175592 | FOSL1 | 163.165163 | 1.90149707 | 0.53559949 | 3.55022195 | 0.0004 | 0.0237 |
| ENSG00000164007 | CLDN19 | 1843.71589 | 2.37953905 | 0.67129051 | 3.54472319 | 0.0004 | 0.0240 |
| ENSG00000267280 | TBX2-AS1 | 475.317725 | -1.53903973 | 0.43399018 | -3.54625474 | 0.0004 | 0.0240 |
| ENSG00000244563 | AC006011.1 | 92.4856442 | -4.74062118 | 1.33693384 | -3.54589063 | 0.0004 | 0.0240 |
| ENSG00000148737 | TCF7L2 | 1385.10287 | 2.20373867 | 0.62165181 | 3.54497267 | 0.0004 | 0.0240 |
| ENSG00000106991 | ENG | 16895.5736 | 1.30729021 | 0.36907874 | 3.54203603 | 0.0004 | 0.0242 |
| ENSG00000156966 | B3GNT7 | 1463.73156 | 2.67613774 | 0.75574255 | 3.54107062 | 0.0004 | 0.0242 |
| ENSG00000227397 | AC079355.2 | 6.53092282 | -6.0950149 | 1.72204605 | -3.53940297 | 0.0004 | 0.0242 |
| ENSG00000010278 | CD9 | 3964.82697 | 1.69109893 | 0.47780841 | 3.53928244 | 0.0004 | 0.0242 |
| ENSG00000225362 | CT62 | 7.30976051 | 5.66112087 | 1.60050395 | 3.53708647 | 0.0004 | 0.0244 |
| ENSG00000169242 | EFNA1 | 4155.97562 | 1.34798542 | 0.38155534 | 3.53287003 | 0.0004 | 0.0247 |

| | | | | | | | |
|---|---|---|---|---|---|---|---|
| ENSG00000210100 | MT-TI | 96.436819 | 2.10113617 | 0.59493094 | 3.53173119 | 0.0004 | 0.0248 |
| ENSG00000121753 | ADGRB2 | 713.748811 | 1.14122684 | 0.32355971 | 3.52709814 | 0.0004 | 0.0251 |
| ENSG00000170482 | SLC23A1 | 29.7616822 | -2.18271506 | 0.6195416 | -3.523113 | 0.0004 | 0.0255 |
| ENSG00000164692 | COL1A2 | 55436.2241 | 1.52362704 | 0.4328587 | 3.51991781 | 0.0004 | 0.0257 |
| ENSG00000229065 | AL354893.2 | 10.3501514 | -6.09243278 | 1.73101555 | -3.51957138 | 0.0004 | 0.0257 |
| ENSG00000154451 | GBP5 | 477.009666 | -2.21794531 | 0.63128756 | -3.5133677 | 0.0004 | 0.0262 |
| ENSG00000273271 | AP000254.1 | 109.130135 | -2.08646242 | 0.59415802 | -3.51162879 | 0.0004 | 0.0263 |
| ENSG00000139304 | PTPRQ | 141.69927 | 1.85292975 | 0.52787583 | 3.51016211 | 0.0004 | 0.0264 |
| ENSG00000273055 | AC005046.1 | 118.393563 | -1.8360437 | 0.52361379 | -3.50648459 | 0.0005 | 0.0266 |
| ENSG00000158445 | KCNB1 | 68.2701214 | 2.98365184 | 0.85134318 | 3.50464058 | 0.0005 | 0.0266 |
| ENSG00000068615 | REEP1 | 290.160093 | 1.663245 | 0.4747072 | 3.50372822 | 0.0005 | 0.0266 |
| ENSG00000174136 | RGMB | 800.894305 | 0.80083029 | 0.22859852 | 3.50321723 | 0.0005 | 0.0266 |
| ENSG00000095752 | IL11 | 171.001354 | 3.00143914 | 0.85668743 | 3.50354054 | 0.0005 | 0.0266 |
| ENSG00000270093 | | 6.16471106 | -6.28131885 | 1.79224077 | -3.50472937 | 0.0005 | 0.0266 |
| ENSG00000114439 | BBX | 1701.68093 | 1.83008343 | 0.52206822 | 3.50544882 | 0.0005 | 0.0266 |
| ENSG00000100292 | HMOX1 | 5934.7016 | 0.91169256 | 0.25994193 | 3.50729319 | 0.0005 | 0.0266 |
| ENSG00000257509 | AC073487.1 | 15.3141526 | -3.56567693 | 1.01912782 | -3.4987534 | 0.0005 | 0.0269 |
| ENSG00000271646 | AC099343.3 | 276.491987 | -1.01346241 | 0.28963621 | -3.49908741 | 0.0005 | 0.0269 |
| ENSG00000182732 | RGS6 | 131.733647 | -2.14251897 | 0.61239801 | -3.49857269 | 0.0005 | 0.0269 |
| ENSG00000151151 | IPMK | 1433.19046 | 1.19414101 | 0.34151169 | 3.4966329 | 0.0005 | 0.0270 |
| ENSG00000273302 | AC016747.3 | 24.2838616 | -2.91654267 | 0.83459596 | -3.49455641 | 0.0005 | 0.0270 |
| ENSG00000269439 | AC010618.3 | 82.7503932 | -1.34496604 | 0.38485285 | -3.49475402 | 0.0005 | 0.0270 |
| ENSG00000107130 | NCS1 | 220.875814 | 1.67985321 | 0.48073984 | 3.4943083 | 0.0005 | 0.0270 |
| ENSG00000070756 | PABPC1 | 11921.9451 | 0.7873476 | 0.22533179 | 3.4941701 | 0.0005 | 0.0270 |
| ENSG00000249669 | CARMN | 3376.33531 | -1.66097327 | 0.47548904 | -3.4931894 | 0.0005 | 0.0271 |
| ENSG00000085741 | WNT11 | 361.21447 | 1.74798057 | 0.5014373 | 3.48594044 | 0.0005 | 0.0277 |
| ENSG00000233251 | AC007743.1 | 1895.20247 | -1.29444432 | 0.37138387 | -3.4854619 | 0.0005 | 0.0277 |
| ENSG00000224452 | RSL24D1P6 | 8.64607895 | -5.34931052 | 1.53503185 | -3.48482054 | 0.0005 | 0.0278 |
| ENSG00000164061 | BSN | 152.365909 | -1.34610322 | 0.38648388 | -3.48294788 | 0.0005 | 0.0279 |
| ENSG00000138755 | CXCL9 | 821.810052 | -5.38484946 | 1.547286 | -3.48019012 | 0.0005 | 0.0281 |
| ENSG00000130598 | TNNI2 | 412.705287 | 2.13198381 | 0.61286173 | 3.47873543 | 0.0005 | 0.0282 |
| ENSG00000170089 | AC106795.1 | 329.40407 | 1.46285338 | 0.42064906 | 3.47760998 | 0.0005 | 0.0283 |
| ENSG00000204172 | AGAP9 | 951.477986 | -1.43642568 | 0.41375324 | -3.47169653 | 0.0005 | 0.0288 |
| ENSG00000159251 | ACTC1 | 182.279643 | 2.22622167 | 0.64116441 | 3.47215413 | 0.0005 | 0.0288 |
| ENSG00000258521 | AL157871.2 | 50.625821 | -2.07685716 | 0.5985456 | -3.46983946 | 0.0005 | 0.0288 |
| ENSG00000198888 | MT-ND1 | 260849.228 | 1.79929936 | 0.51853557 | 3.4699632 | 0.0005 | 0.0288 |
| ENSG00000269514 | AC024257.3 | 70.9170663 | -2.12854493 | 0.61356983 | -3.46911603 | 0.0005 | 0.0288 |
| ENSG00000007171 | NOS2 | 133.379018 | 2.78435415 | 0.80361368 | 3.4647919 | 0.0005 | 0.0293 |
| ENSG00000149925 | ALDOA | 20322.0633 | 0.78603866 | 0.22698281 | 3.46298765 | 0.0005 | 0.0294 |
| ENSG00000198393 | ZNF26 | 947.468478 | -0.78341985 | 0.2269931 | -3.45129363 | 0.0006 | 0.0306 |
| ENSG00000132164 | SLC6A11 | 111.830809 | 2.38776071 | 0.6920495 | 3.45027446 | 0.0006 | 0.0306 |
| ENSG00000115310 | RTN4 | 12085.1087 | 0.71710417 | 0.20780889 | 3.45078672 | 0.0006 | 0.0306 |
| ENSG00000165474 | GJB2 | 15.1096156 | 4.11298243 | 1.19298474 | 3.44764044 | 0.0006 | 0.0308 |
| ENSG00000255571 | MIR9-3HG | 202.604666 | 2.12849587 | 0.61770164 | 3.44583165 | 0.0006 | 0.0309 |
| ENSG00000280434 | AL031595.3 | 95.3674667 | -2.30842445 | 0.66980924 | -3.44639087 | 0.0006 | 0.0309 |
| ENSG00000167113 | COQ4 | 2300.23688 | -0.56886558 | 0.16511375 | -3.445295 | 0.0006 | 0.0309 |
| ENSG00000110218 | PANX1 | 820.062602 | 1.17868156 | 0.34228625 | 3.44355509 | 0.0006 | 0.0310 |
| ENSG00000125851 | PCSK2 | 25.5535196 | 5.55360856 | 1.61366237 | 3.44161745 | 0.0006 | 0.0312 |
| ENSG00000092621 | PHGDH | 798.233491 | 1.16061643 | 0.3374027 | 3.4398552 | 0.0006 | 0.0312 |
| ENSG00000134343 | ANO3 | 64.3220915 | 2.72769835 | 0.79288081 | 3.44023755 | 0.0006 | 0.0312 |
| ENSG00000274008 | RF00017 | 30.0999274 | -2.40958403 | 0.70049284 | -3.43984103 | 0.0006 | 0.0312 |
| ENSG00000229376 | CICP3 | 22.6049306 | -2.15371702 | 0.62672441 | -3.43646581 | 0.0006 | 0.0315 |
| ENSG00000156273 | BACH1 | 3908.83812 | 1.23835377 | 0.3605189 | 3.43492053 | 0.0006 | 0.0316 |
| ENSG00000116183 | PAPPA2 | 105464.035 | 2.48018513 | 0.72419319 | 3.42475621 | 0.0006 | 0.0324 |
| ENSG00000186104 | CYP2R1 | 251.372311 | -0.84903897 | 0.24787105 | -3.42532529 | 0.0006 | 0.0324 |
| ENSG00000230156 | LINC00443 | 7.94156057 | -6.54348755 | 1.91073138 | -3.42459835 | 0.0006 | 0.0324 |
| ENSG00000100934 | SEC23A | 2350.58811 | 0.81706941 | 0.23848791 | 3.42604128 | 0.0006 | 0.0324 |
| ENSG00000215067 | ALOX12-AS1 | 247.468614 | -1.2172132 | 0.35548634 | -3.42407872 | 0.0006 | 0.0324 |
| ENSG00000099834 | CDHR5 | 109.105416 | -1.93717141 | 0.56565218 | -3.42466887 | 0.0006 | 0.0324 |
| ENSG00000262319 | AC007952.6 | 121.893391 | -1.83700323 | 0.53612183 | -3.42646598 | 0.0006 | 0.0324 |
| ENSG00000088298 | EDEM2 | 2935.45497 | 0.62355036 | 0.18215141 | 3.42325297 | 0.0006 | 0.0325 |
| ENSG00000229052 | AL449283.1 | 6.9764123 | -6.38703774 | 1.86881717 | -3.41768999 | 0.0006 | 0.0331 |
| ENSG00000198712 | MT-CO2 | 260525.838 | 1.30018871 | 0.38094358 | 3.41307425 | 0.0006 | 0.0336 |
| ENSG00000136918 | WDR38 | 6.91366459 | 4.9437412 | 1.45042426 | 3.40847938 | 0.0007 | 0.0340 |
| ENSG00000198865 | CCDC152 | 609.603887 | -1.46746857 | 0.43057412 | -3.40816713 | 0.0007 | 0.0340 |
| ENSG00000146242 | TPBG | 4180.48987 | 1.50334076 | 0.44119374 | 3.40743901 | 0.0007 | 0.0341 |
| ENSG00000238140 | AC104170.2 | 5.95567186 | -5.79408846 | 1.70170996 | -3.40486253 | 0.0007 | 0.0343 |
| ENSG00000100739 | BDKRB1 | 60.0361074 | 2.78611172 | 0.8183768 | 3.40443634 | 0.0007 | 0.0343 |
| ENSG00000151292 | CSNK1G3 | 615.094323 | 1.22621097 | 0.36026645 | 3.40362239 | 0.0007 | 0.0343 |
| ENSG00000114812 | VIPR1 | 545.072474 | -2.08799271 | 0.61377963 | -3.40186057 | 0.0007 | 0.0345 |
| ENSG00000205413 | SAMD9 | 851.293503 | 1.10514329 | 0.32512901 | 3.39909162 | 0.0007 | 0.0348 |

| | | | | | | | |
|---|---|---|---|---|---|---|---|
| ENSG00000274653 | AC106782.6 | 71.1486805 | -1.4539952 | 0.42802584 | -3.39697998 | 0.0007 | 0.0350 |
| ENSG00000248124 | RRN3P1 | 656.341035 | -0.95593811 | 0.28165485 | -3.39400547 | 0.0007 | 0.0350 |
| ENSG00000171097 | KYAT1 | 337.512171 | 1.20169714 | 0.35393733 | 3.39522573 | 0.0007 | 0.0350 |
| ENSG00000160789 | LMNA | 17610.0267 | 0.72151475 | 0.21245067 | 3.39615184 | 0.0007 | 0.0350 |
| ENSG00000146021 | KLHL3 | 1007.50174 | -0.96475697 | 0.28417236 | -3.39497122 | 0.0007 | 0.0350 |
| ENSG00000143217 | NECTIN4 | 12871.5096 | 1.52839556 | 0.45032833 | 3.3939583 | 0.0007 | 0.0350 |
| ENSG00000111640 | GAPDH | 57384.1053 | 1.06510873 | 0.31400447 | 3.39201777 | 0.0007 | 0.0351 |
| ENSG00000138316 | ADAMTS14 | 1336.80947 | -1.01499489 | 0.29927191 | -3.39154747 | 0.0007 | 0.0351 |
| ENSG00000142798 | HSPG2 | 40709.761 | 1.73181512 | 0.51054216 | 3.39210989 | 0.0007 | 0.0351 |
| ENSG00000119314 | PTBP3 | 4517.44426 | 1.00791935 | 0.29730417 | 3.39019574 | 0.0007 | 0.0352 |
| ENSG00000131981 | LGALS3 | 5685.32956 | 1.55917717 | 0.46019592 | 3.38807255 | 0.0007 | 0.0354 |
| ENSG00000139973 | SYT16 | 298.54514 | 1.93527022 | 0.57156861 | 3.38589309 | 0.0007 | 0.0356 |
| ENSG00000145287 | PLAC8 | 1177.70899 | 2.27776912 | 0.67292375 | 3.38488444 | 0.0007 | 0.0357 |
| ENSG00000124766 | SOX4 | 1597.30917 | 1.51025429 | 0.44630088 | 3.3839375 | 0.0007 | 0.0357 |
| ENSG00000089154 | GCN1 | 9610.69574 | 0.76485178 | 0.22609837 | 3.38282747 | 0.0007 | 0.0357 |
| ENSG00000080947 | CROCCP3 | 472.652029 | -1.01254783 | 0.29928883 | -3.38317946 | 0.0007 | 0.0357 |
| ENSG00000184347 | SLIT3 | 3348.96928 | 1.27998911 | 0.3787948 | 3.37910949 | 0.0007 | 0.0361 |
| ENSG00000119514 | GALNT12 | 216.528136 | 1.28027913 | 0.37916928 | 3.37653709 | 0.0007 | 0.0363 |
| ENSG00000270039 | AC025165.5 | 56.2141696 | -1.63171468 | 0.48325621 | -3.37650017 | 0.0007 | 0.0363 |
| ENSG00000169851 | PCDH7 | 36.5519853 | 4.06743463 | 1.2060236 | 3.37259955 | 0.0007 | 0.0368 |
| ENSG00000267747 | AC068675.1 | 6.60604182 | -5.72175358 | 1.69666815 | -3.3723469 | 0.0007 | 0.0368 |
| ENSG00000213693 | SEC14L1P1 | 45.5645218 | -2.41538416 | 0.7169542 | -3.36895182 | 0.0008 | 0.0371 |
| ENSG00000168214 | RBPJ | 4410.42375 | -1.41372984 | 0.41988619 | -3.36693579 | 0.0008 | 0.0373 |
| ENSG00000124145 | SDC4 | 4792.77679 | 1.45146698 | 0.43168626 | 3.36231914 | 0.0008 | 0.0378 |
| ENSG00000164403 | SHROOM1 | 3039.41909 | -1.24241971 | 0.36950555 | -3.36238449 | 0.0008 | 0.0378 |
| ENSG00000140398 | NEIL1 | 1295.69845 | -0.72622054 | 0.21608767 | -3.360768 | 0.0008 | 0.0380 |
| ENSG00000226252 | AL135960.1 | 341.919594 | -1.45142913 | 0.43225922 | -3.35777482 | 0.0008 | 0.0383 |
| ENSG00000102287 | GABRE | 30197.1572 | -1.13959663 | 0.33991869 | -3.35255659 | 0.0008 | 0.0388 |
| ENSG00000232070 | TMEM253 | 17.0359003 | -2.82390655 | 0.842051 | -3.35360512 | 0.0008 | 0.0388 |
| ENSG00000272180 | AC011306.1 | 137.081052 | -1.96884523 | 0.58722233 | -3.35281058 | 0.0008 | 0.0388 |
| ENSG00000140416 | TPM1 | 16737.235 | 1.47042836 | 0.43867397 | 3.35198451 | 0.0008 | 0.0388 |
| ENSG00000254245 | PCDHGA3 | 304.045147 | 2.24036659 | 0.66882474 | 3.34970651 | 0.0008 | 0.0390 |
| ENSG00000140743 | CDR2 | 1108.71213 | 0.84832657 | 0.25322438 | 3.35009831 | 0.0008 | 0.0390 |
| ENSG00000166448 | TMEM130 | 34.4870983 | 3.72440731 | 1.1122086 | 3.34865899 | 0.0008 | 0.0391 |
| ENSG00000115677 | HDLBP | 18217.9424 | 0.67917754 | 0.2031384 | 3.34342266 | 0.0008 | 0.0393 |
| ENSG00000242371 | IGKV1-39 | 12.0585612 | -7.8210602 | 2.33952677 | -3.34300949 | 0.0008 | 0.0393 |
| ENSG00000279900 | AP001767.4 | 8.16862238 | -6.3140361 | 1.88734726 | -3.34545541 | 0.0008 | 0.0393 |
| ENSG00000260139 | CSPG4P13 | 73.1269581 | -1.89408859 | 0.56649872 | -3.34350023 | 0.0008 | 0.0393 |
| ENSG00000107249 | GLIS3 | 313.215645 | 1.32736869 | 0.3966887 | 3.34612173 | 0.0008 | 0.0393 |
| ENSG00000151303 | AL136982.1 | 105.464115 | -4.03858531 | 1.20776591 | -3.34384775 | 0.0008 | 0.0393 |
| ENSG00000122085 | MTERF4 | 1141.42785 | -0.56894491 | 0.1701292 | -3.34419314 | 0.0008 | 0.0393 |
| ENSG00000166343 | MSS51 | 121.413053 | -1.79447447 | 0.53622188 | -3.34651484 | 0.0008 | 0.0393 |
| ENSG00000211893 | IGHG2 | 232.399445 | -2.49449321 | 0.74639868 | -3.34203863 | 0.0008 | 0.0393 |
| ENSG00000145147 | SLIT2 | 1280.20974 | 1.29927142 | 0.38892165 | 3.34070225 | 0.0008 | 0.0394 |
| ENSG00000203870 | SMIM9 | 14.8333813 | -4.72889706 | 1.41532957 | -3.34119851 | 0.0008 | 0.0394 |
| ENSG00000181751 | C5orf30 | 833.138669 | 1.25915921 | 0.37708593 | 3.33918375 | 0.0008 | 0.0394 |
| ENSG00000127325 | BEST3 | 15.7384683 | 4.92901289 | 1.47607372 | 3.33927285 | 0.0008 | 0.0394 |
| ENSG00000114268 | PFKFB4 | 1784.07256 | 0.98508299 | 0.29517594 | 3.33727398 | 0.0008 | 0.0396 |
| ENSG00000225399 | AC121247.1 | 38.0950132 | -2.28598429 | 0.68499834 | -3.33721144 | 0.0008 | 0.0396 |
| ENSG00000229142 | HCG4P8 | 836.480515 | 3.70742588 | 1.11246018 | 3.33263694 | 0.0009 | 0.0400 |
| ENSG00000103089 | FA2H | 75.8182243 | 3.27262229 | 0.98208661 | 3.33231535 | 0.0009 | 0.0400 |
| ENSG00000164438 | TLX3 | 13.0827297 | 4.58697695 | 1.3764113 | 3.33256269 | 0.0009 | 0.0400 |
| ENSG00000246366 | LACTB2-AS1 | 6.64118677 | -6.05309529 | 1.81730925 | -3.3308009 | 0.0009 | 0.0401 |
| ENSG00000215105 | TTC3P1 | 85.5805371 | 1.15664917 | 0.3472172 | 3.33119779 | 0.0009 | 0.0401 |
| ENSG00000280152 | AC009078.3 | 436.56796 | -1.30357618 | 0.39201667 | -3.32530803 | 0.0009 | 0.0408 |
| ENSG00000116285 | ERRFI1 | 18328.6058 | 1.01955589 | 0.30672866 | 3.32396679 | 0.0009 | 0.0410 |
| ENSG00000105290 | APLP1 | 76.2649899 | 2.69216058 | 0.81010171 | 3.32323775 | 0.0009 | 0.0410 |
| ENSG00000150764 | DIXDC1 | 1586.114 | 0.99129138 | 0.29854078 | 3.32045548 | 0.0009 | 0.0413 |
| ENSG00000245849 | RAD51-AS1 | 440.957952 | -1.34642985 | 0.40559132 | -3.31967123 | 0.0009 | 0.0414 |
| ENSG00000272486 | AC090922.1 | 23.7293942 | -2.66729817 | 0.80447784 | -3.31556451 | 0.0009 | 0.0419 |
| ENSG00000109586 | GALNT7 | 1036.57866 | 1.21137048 | 0.36548963 | 3.31437719 | 0.0009 | 0.0420 |
| ENSG00000235865 | GSN-AS1 | 270.328016 | -1.19598193 | 0.36092555 | -3.31365273 | 0.0009 | 0.0420 |
| ENSG00000165617 | DACT1 | 831.066937 | 1.18942354 | 0.35899305 | 3.31322161 | 0.0009 | 0.0420 |
| ENSG00000228742 | LINC02577 | 31.343336 | 2.33126558 | 0.70390698 | 3.31189441 | 0.0009 | 0.0421 |
| ENSG00000145675 | PIK3R1 | 2384.94 | 1.30870649 | 0.39511815 | 3.31219029 | 0.0009 | 0.0421 |
| ENSG00000053747 | LAMA3 | 7241.17832 | 1.98418723 | 0.59948625 | 3.30981275 | 0.0009 | 0.0423 |
| ENSG00000223901 | AP001469.1 | 17.2163667 | -2.98585279 | 0.90253271 | -3.30830424 | 0.0009 | 0.0424 |
| ENSG00000281344 | HELLPAR | 153.101361 | -2.02103452 | 0.61093869 | -3.30808075 | 0.0009 | 0.0424 |
| ENSG00000198763 | MT-ND2 | 340928.29 | 1.68985155 | 0.5112076 | 3.30560722 | 0.0009 | 0.0427 |
| ENSG00000267281 | AC023509.3 | 341.706676 | -1.10844184 | 0.33563558 | -3.30251591 | 0.0010 | 0.0431 |
| ENSG00000101945 | SUV39H1 | 426.621471 | 1.54782082 | 0.46902298 | 3.30009595 | 0.0010 | |

| | | | | | | | |
|---|---|---|---|---|---|---|---|
| ENSG00000122884 | P4HA1 | 3189.23132 | 1.19148615 | 0.36117973 | 3.29887327 | 0.0010 | 0.0435 |
| ENSG00000149634 | SPATA25 | 50.2351053 | -1.38746364 | 0.42086237 | -3.29671584 | 0.0010 | 0.0438 |
| ENSG00000271851 | AC087501.4 | 99.218647 | -1.65079236 | 0.5009025 | -3.29563613 | 0.0010 | 0.0439 |
| ENSG00000144837 | PLA1A | 543.314665 | -1.4103425 | 0.4281311 | -3.29418375 | 0.0010 | 0.0440 |
| ENSG00000205918 | PDPK2P | 212.820537 | -1.39871031 | 0.42466593 | -3.2936721 | 0.0010 | 0.0440 |
| ENSG00000108219 | TSPAN14 | 7802.30037 | 0.74628431 | 0.22668199 | 3.29220823 | 0.0010 | 0.0442 |
| ENSG00000054277 | OPN3 | 2616.77288 | 1.36771011 | 0.41549376 | 3.29177053 | 0.0010 | 0.0442 |
| ENSG00000084636 | COL16A1 | 4714.64733 | 1.58502176 | 0.48188687 | 3.28919887 | 0.0010 | 0.0445 |
| ENSG00000157404 | KIT | 704.565 | 2.02112261 | 0.61478788 | 3.28751213 | 0.0010 | 0.0447 |
| ENSG00000270110 | AL353593.3 | 13.4794427 | -3.2885164 | 1.00047345 | -3.2869602 | 0.0010 | 0.0447 |
| ENSG00000272599 | AC016394.2 | 61.458944 | -1.582571 | 0.48211846 | -3.28253556 | 0.0010 | 0.0453 |
| ENSG00000268297 | CLEC4GP1 | 221.856633 | -2.16788909 | 0.6606774 | -3.28131263 | 0.0010 | 0.0454 |
| ENSG00000279339 | AC100788.2 | 112.409233 | -2.13066044 | 0.64984155 | -3.2787384 | 0.0010 | 0.0458 |
| ENSG00000236383 | LINC00854 | 95.8748368 | -1.32553605 | 0.40460887 | -3.2760924 | 0.0011 | 0.0461 |
| ENSG00000283378 | BX088645.1 | 107.84191 | 2.83533475 | 0.86536668 | 3.27645473 | 0.0011 | 0.0461 |
| ENSG00000003137 | CYP26B1 | 966.522942 | 1.46693995 | 0.44824959 | 3.27259626 | 0.0011 | 0.0465 |
| ENSG00000279573 | AC134407.2 | 22.9668621 | -3.237424 | 0.98980859 | -3.27075763 | 0.0011 | 0.0466 |
| ENSG00000197670 | AL157838.1 | 104.192785 | -1.33595261 | 0.40842938 | -3.27095128 | 0.0011 | 0.0466 |
| ENSG00000283646 | LINC02009 | 40.849558 | 3.91899805 | 1.19789448 | 3.271572 | 0.0011 | 0.0466 |
| ENSG00000210107 | MT-TQ | 32.5202952 | 2.45040187 | 0.74955539 | 3.2691405 | 0.0011 | 0.0467 |
| ENSG00000173451 | THAP2 | 265.929128 | 1.5030646 | 0.45980989 | 3.26888273 | 0.0011 | 0.0467 |
| ENSG00000197956 | S100A6 | 16267.2414 | 1.04187888 | 0.31872678 | 3.2688777 | 0.0011 | 0.0467 |
| ENSG00000268471 | MIR4453HG | 284.283888 | -1.01891415 | 0.31188399 | -3.26696525 | 0.0011 | 0.0468 |
| ENSG00000260279 | AC137932.1 | 29.5088386 | -1.72916222 | 0.5293323 | -3.26668561 | 0.0011 | 0.0468 |
| ENSG00000219607 | PPP1R3G | 293.502659 | 1.3738051 | 0.42054355 | 3.26673679 | 0.0011 | 0.0468 |
| ENSG00000167553 | TUBA1C | 6723.70212 | 0.71225619 | 0.21825869 | 3.26335779 | 0.0011 | 0.0468 |
| ENSG00000198899 | MT-ATP6 | 390198.956 | 1.35275428 | 0.41453895 | 3.26327422 | 0.0011 | 0.0468 |
| ENSG00000128266 | GNAZ | 71.1650117 | 1.47078504 | 0.45069262 | 3.26338832 | 0.0011 | 0.0468 |
| ENSG00000121690 | DEPDC7 | 75.2346109 | 2.0235816 | 0.62011277 | 3.26324775 | 0.0011 | 0.0468 |
| ENSG00000199622 | RN7SKP20 | 6.23240072 | -6.13226027 | 1.87805034 | -3.26522679 | 0.0011 | 0.0468 |
| ENSG00000158516 | CPA2 | 15.5470547 | -5.17924885 | 1.58622819 | -3.2651348 | 0.0011 | 0.0468 |
| ENSG00000280157 | AL359510.2 | 89.2649091 | -1.59179146 | 0.4874546 | -3.26551736 | 0.0011 | 0.0468 |
| ENSG00000225630 | MTND2P28 | 42205.2672 | 1.69459727 | 0.51946195 | 3.26221638 | 0.0011 | 0.0469 |
| ENSG00000268516 | AC020915.3 | 236.929197 | -0.99956122 | 0.30642121 | -3.26204975 | 0.0011 | 0.0469 |
| ENSG00000164171 | ITGA2 | 743.460268 | 1.54215424 | 0.47308281 | 3.25979764 | 0.0011 | 0.0469 |
| ENSG00000136068 | FLNB | 19031.743 | 1.48725693 | 0.45611292 | 3.26072094 | 0.0011 | 0.0469 |
| ENSG00000279518 | AC083843.4 | 629.218129 | -1.64497196 | 0.5047246 | -3.25914758 | 0.0011 | 0.0469 |
| ENSG00000138615 | CILP | 348.492396 | 2.06984119 | 0.63501039 | 3.25953912 | 0.0011 | 0.0469 |
| ENSG00000102265 | TIMP1 | 15322.4807 | 1.86165083 | 0.57114284 | 3.25951883 | 0.0011 | 0.0469 |
| ENSG00000269910 | AL049840.2 | 150.740645 | -1.34567649 | 0.41300913 | -3.25822459 | 0.0011 | 0.0470 |
| ENSG00000279384 | AC080188.2 | 19.8716664 | -3.58706801 | 1.10138272 | -3.25687696 | 0.0011 | 0.0471 |
| ENSG00000050327 | ARHGEF5 | 2122.72584 | 0.82056777 | 0.2519217 | 3.25723342 | 0.0011 | 0.0471 |
| ENSG00000197273 | GUCA2A | 339.930137 | 2.07710292 | 0.63804876 | 3.25539843 | 0.0011 | 0.0473 |
| ENSG00000197815 | AC122129.1 | 129.958552 | -1.74777202 | 0.5370073 | -3.25465223 | 0.0011 | 0.0473 |
| ENSG00000279640 | AC138466.5 | 22.5673629 | -3.95352158 | 1.21498035 | -3.25397985 | 0.0011 | 0.0473 |
| ENSG00000196872 | KIAA1211L | 1155.89534 | 1.22308629 | 0.37602824 | 3.25264475 | 0.0011 | 0.0474 |
| ENSG00000259124 | AC008050.1 | 20.7846951 | -2.41840416 | 0.74341795 | -3.25308821 | 0.0011 | 0.0474 |
| ENSG00000146197 | SCUBE3 | 216.415139 | 2.00153106 | 0.61573176 | 3.25065425 | 0.0012 | 0.0474 |
| ENSG00000176871 | WSB2 | 1790.05872 | 0.62751984 | 0.19301872 | 3.25108272 | 0.0011 | 0.0474 |
| ENSG00000184434 | LRRC19 | 6.63668157 | -5.13121186 | 1.57840497 | -3.25088424 | 0.0012 | 0.0474 |
| ENSG00000173511 | VEGFB | 2366.85257 | 0.78043273 | 0.24002936 | 3.25140533 | 0.0011 | 0.0474 |
| ENSG00000197992 | CLEC9A | 29.6509662 | -1.89753165 | 0.58390215 | -3.24974253 | 0.0012 | 0.0475 |
| ENSG00000163902 | RPN1 | 13412.811 | 0.59069073 | 0.18187518 | 3.24778085 | 0.0012 | 0.0477 |
| ENSG00000280302 | AP005264.7 | 19.7483823 | -3.68362774 | 1.13475099 | -3.2461992 | 0.0012 | 0.0479 |
| ENSG00000246273 | SBF2-AS1 | 234.384443 | 1.24831748 | 0.38453888 | 3.24627113 | 0.0012 | 0.0479 |
| ENSG00000135960 | EDAR | 8.50305278 | 5.9409226 | 1.83197839 | 3.24289994 | 0.0012 | 0.0483 |
| ENSG00000279059 | AC007485.2 | 167.789146 | -1.28913068 | 0.39783356 | -3.24037687 | 0.0012 | 0.0487 |
| ENSG00000115267 | IFIH1 | 1126.5291 | 0.9178004 | 0.28333915 | 3.23922906 | 0.0012 | 0.0487 |
| ENSG00000106034 | CPED1 | 1442.61957 | 0.99081694 | 0.30588113 | 3.23922212 | 0.0012 | 0.0487 |
| ENSG00000151458 | ANKRD50 | 1948.29966 | 1.19492687 | 0.36911926 | 3.23723791 | 0.0012 | 0.0490 |
| ENSG00000033867 | SLC4A7 | 1233.31697 | 1.59737553 | 0.49358086 | 3.2362996 | 0.0012 | 0.0491 |
| ENSG00000210195 | MT-TT | 120.361123 | 1.8608977 | 0.57537233 | 3.23424955 | 0.0012 | 0.0492 |
| ENSG00000107954 | NEURL1 | 4730.86334 | 1.65099492 | 0.51032949 | 3.23515483 | 0.0012 | 0.0492 |
| ENSG00000141429 | GALNT1 | 4059.61394 | 0.96045029 | 0.29695121 | 3.23437063 | 0.0012 | 0.0492 |
| ENSG00000091527 | CDV3 | 2942.13844 | 0.88392244 | 0.2733591 | 3.2335578 | 0.0012 | 0.0492 |
| ENSG00000110975 | SYT10 | 9.25764129 | 6.98361222 | 2.1607754 | 3.23199357 | 0.0012 | 0.0494 |
| ENSG00000231858 | AC067945.3 | 7.37677709 | -6.11320113 | 1.89200801 | -3.23106515 | 0.0012 | 0.0495 |
| ENSG00000011465 | DCN | 25194.7252 | 0.79485176 | 0.24607539 | 3.23011482 | 0.0012 | 0.0495 |
| ENSG00000169604 | ANTXR1 | 7311.57394 | 1.26359629 | 0.39118086 | 3.23020994 | 0.0012 | 0.0495 |
| ENSG00000250397 | AP006623.1 | 172.91574 | -1.48891047 | 0.46120309 | -3.22831854 | 0.0012 | 0.0496 |
| ENSG00000267069 | AP005264.1 | 14.1995177 | 3.44077021 | 1.0660212 | 3.22767522 | 0.0012 | 0.0496 |

| | | | | | | | |
|---|---|---|---|---|---|---|---|
| ENSG00000133216 | EPHB2 | 1715.06202 | 1.59561024 | 0.49421724 | 3.22856048 | 0.0012 | 0.0496 |
| ENSG00000154175 | ABI3BP | 1886.17134 | 1.90564523 | 0.59043629 | 3.22752049 | 0.0012 | 0.0496 |
| ENSG00000166669 | ATF7IP2 | 519.622685 | -0.93778535 | 0.29060074 | -3.22705771 | 0.0013 | 0.0496 |
| ENSG00000106789 | CORO2A | 671.888986 | 1.74868487 | 0.54195712 | 3.22661113 | 0.0013 | 0.0496 |
| ENSG00000186148 | AC013268.1 | 1057.94065 | 1.45154909 | 0.4499742 | 3.2258496 | 0.0013 | 0.0497 |
| ENSG00000074410 | CA12 | 293.579775 | 2.44324922 | 0.75759599 | 3.22500285 | 0.0013 | 0.0498 |

***S4c. All differentially expressed genes found for the model design with 4 conditions (male control, male SSRI, female control, and female SSRI), contrasted for females, comparing female control, and female fluoxetine.*** lfcSE*: standard error of the log2FoldChange.* Stat*: the Wald statistic for the Wald test, which is compared to a standard Normal distribution to generate a two-tailed* p-value. Padj: *p-value adjusted for multiple testing using the Benjamini-Hochberg method.*

| ENSEMBL ID | Gene Symbol | baseMean | log2FoldChange | lfcSE | stat | pvalue | padj |
|---|---|---|---|---|---|---|---|
| ENSG00000260342 | AC138811.2 | 295.361365 | -33.6126653 | 4.6002077 | -7.30677124 | 0.0000 | 0.0000 |
| ENSG00000124208 | TMEM189-UBE2V1 | 297.721703 | 29.9466585 | 4.5240468 | 6.61944048 | 0.0000 | 0.0000 |
| ENSG00000267904 | AC024075.1 | 21.9153032 | -29.5365512 | 4.60253802 | -6.4174486 | 0.0000 | 0.0000 |
| ENSG00000273373 | AL355488.1 | 470.629645 | 1.66847192 | 0.26446484 | 6.30886102 | 0.0000 | 0.0000 |
| ENSG00000160712 | IL6R | 670.16632 | -4.20761307 | 0.74596002 | -5.64053432 | 0.0000 | 0.0001 |
| ENSG00000254870 | ATP6V1G2-DDX39B | 318.276138 | -25.7737988 | 4.60012203 | -5.6028511 | 0.0000 | 0.0001 |
| ENSG00000261326 | LINC01355 | 1031.34719 | 1.77787288 | 0.32488629 | 5.47229273 | 0.0000 | 0.0002 |
| ENSG00000213918 | DNASE1 | 2428.26774 | 0.95969696 | 0.19005951 | 5.0494552 | 0.0000 | 0.0014 |
| ENSG00000171067 | C11orf24 | 1062.31595 | 1.11736135 | 0.22532043 | 4.95898819 | 0.0000 | 0.0020 |
| ENSG00000135503 | ACVR1B | 1192.71321 | -2.01279432 | 0.41090429 | -4.89845053 | 0.0000 | 0.0024 |
| ENSG00000176020 | AMIGO3 | 332.29035 | -4.5390929 | 0.93017291 | -4.87983779 | 0.0000 | 0.0024 |
| ENSG00000251349 | MSANTD3-TMEFF1 | 41.7570487 | 20.663024 | 4.33068633 | 4.77130469 | 0.0000 | 0.0039 |
| ENSG00000280067 | AC023818.1 | 114.513668 | 1.75427827 | 0.37347912 | 4.69712544 | 0.0000 | 0.0051 |
| ENSG00000005381 | MPO | 49.8266269 | -2.88686355 | 0.62825933 | -4.59501902 | 0.0000 | 0.0074 |
| ENSG00000251357 | AP000350.4 | 25.9173057 | -21.1253696 | 4.60116789 | -4.59130596 | 0.0000 | 0.0074 |
| ENSG00000259972 | AC009120.2 | 371.974094 | 1.93370205 | 0.42501167 | 4.54976229 | 0.0000 | 0.0085 |
| ENSG00000152332 | UHMK1 | 3277.69282 | -2.16647766 | 0.48357919 | -4.48008873 | 0.0000 | 0.0105 |
| ENSG00000118520 | ARG1 | 41.3063947 | -7.24953957 | 1.6173144 | -4.48245534 | 0.0000 | 0.0105 |
| ENSG00000160392 | C19orf47 | 815.467915 | -1.4819972 | 0.33294015 | -4.45124204 | 0.0000 | 0.0113 |
| ENSG00000131374 | TBC1D5 | 2080.12969 | -1.44341927 | 0.32882068 | -4.38968525 | 0.0000 | 0.0143 |
| ENSG00000267934 | AC010300.1 | 406.805583 | 2.11138833 | 0.48337112 | 4.36804815 | 0.0000 | 0.0151 |
| ENSG00000132703 | APCS | 28.7519789 | -8.41972641 | 1.95852996 | -4.29900312 | 0.0000 | 0.0197 |
| ENSG00000127366 | TAS2R5 | 64.0684438 | 2.65707118 | 0.62575749 | 4.24616763 | 0.0000 | 0.0239 |
| ENSG00000183878 | UTY | 624.081774 | -6.74500717 | 1.62787014 | -4.1434553 | 0.0000 | 0.0360 |
| ENSG00000186951 | PPARA | 626.872599 | 1.69374673 | 0.41003157 | 4.13077154 | 0.0000 | 0.0365 |
| ENSG00000248329 | APELA | 21.3084687 | -7.72200276 | 1.87546226 | -4.11738637 | 0.0000 | 0.0372 |
| ENSG00000149346 | SLX4IP | 437.019152 | 1.06675883 | 0.26049336 | 4.0951479 | 0.0000 | 0.0395 |
| ENSG00000271430 | | 1516.49252 | 0.94849689 | 0.235141 | 4.0337368 | 0.0001 | 0.0495 |
| ENSG00000240979 | AL353813.1 | 23.8764535 | 5.02454773 | 1.25365421 | 4.00792154 | 0.0001 | 0.0499 |
| ENSG00000253196 | AC083841.1 | 13.193454 | 6.17409807 | 1.53788858 | 4.0146589 | 0.0001 | 0.0499 |
| ENSG00000124191 | TOX2 | 296.056779 | -1.98182815 | 0.49342319 | -4.01648767 | 0.0001 | 0.0499 |

***S5. Network of enriched terms associated with fluoxetine treatment in male and female placenta.*** *Each node represents an enriched term and, where its size is proportional to the number of differentially expressed genes that fall into the term. The nodes are represented as pie-charts, color-coded for whether the identities of the gene lists came from the DEGs in male placenta (blue) or female placenta (red). Only very few nodes were overrepresented in the DEGs of both male and female placentas (indicated by grey arrow head).*

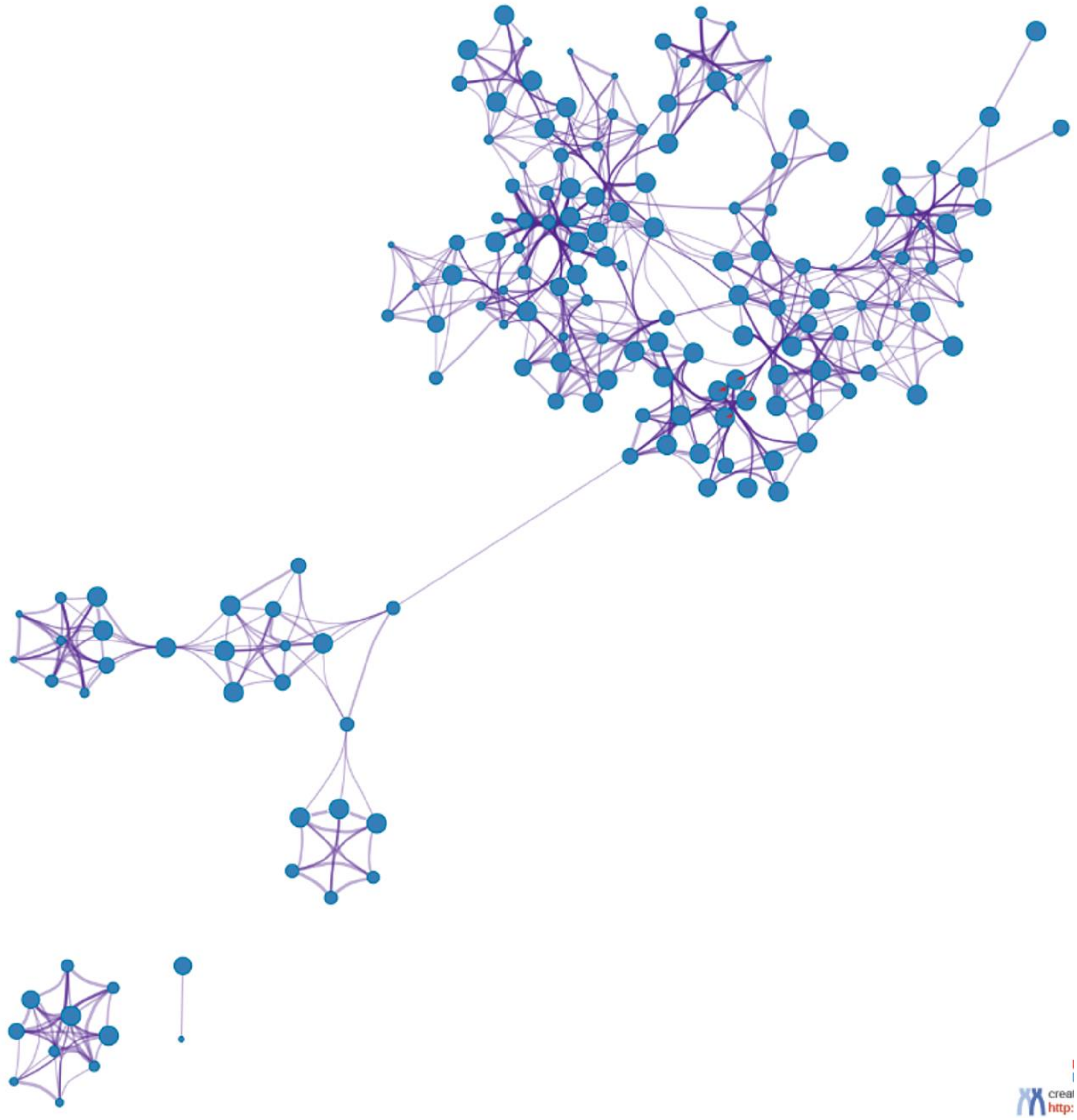

***S6. Network of enriched terms associated with fluoxetine treatment in male and female placenta.*** *Nodes are color-coded by p-value, where terms containing more genes tend to have a more significant p-value. Each node represents an enriched term and, where its size is proportional to the number of differentially expressed genes that fall into the term.*

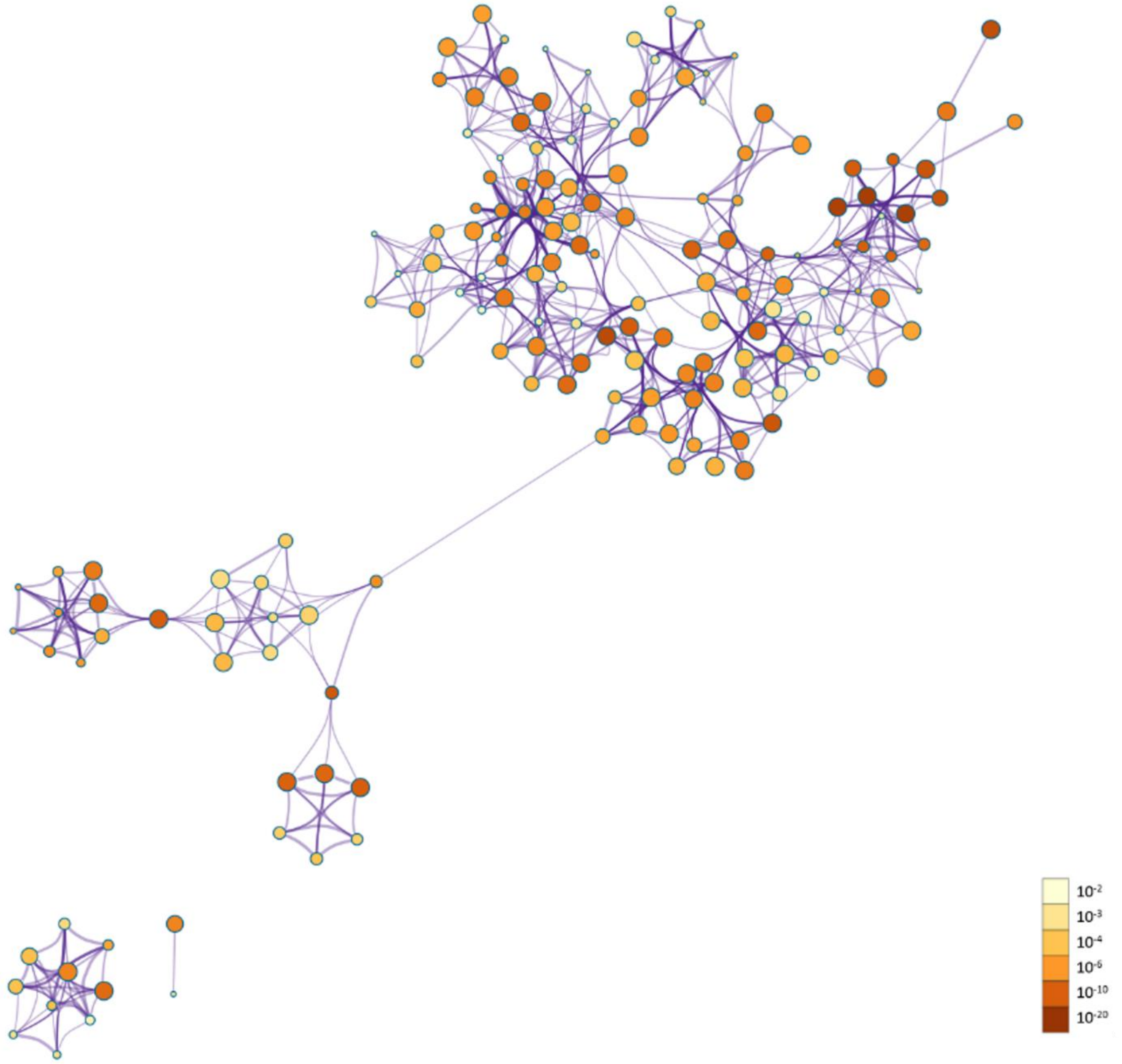

*S7.**Heatmap of top 100 enriched terms across input gene lists, coloured by p-values.** Grey cells indicate the lack of enrichment for that term in the corresponding gene list. The darker the colour, the more statistically significant*

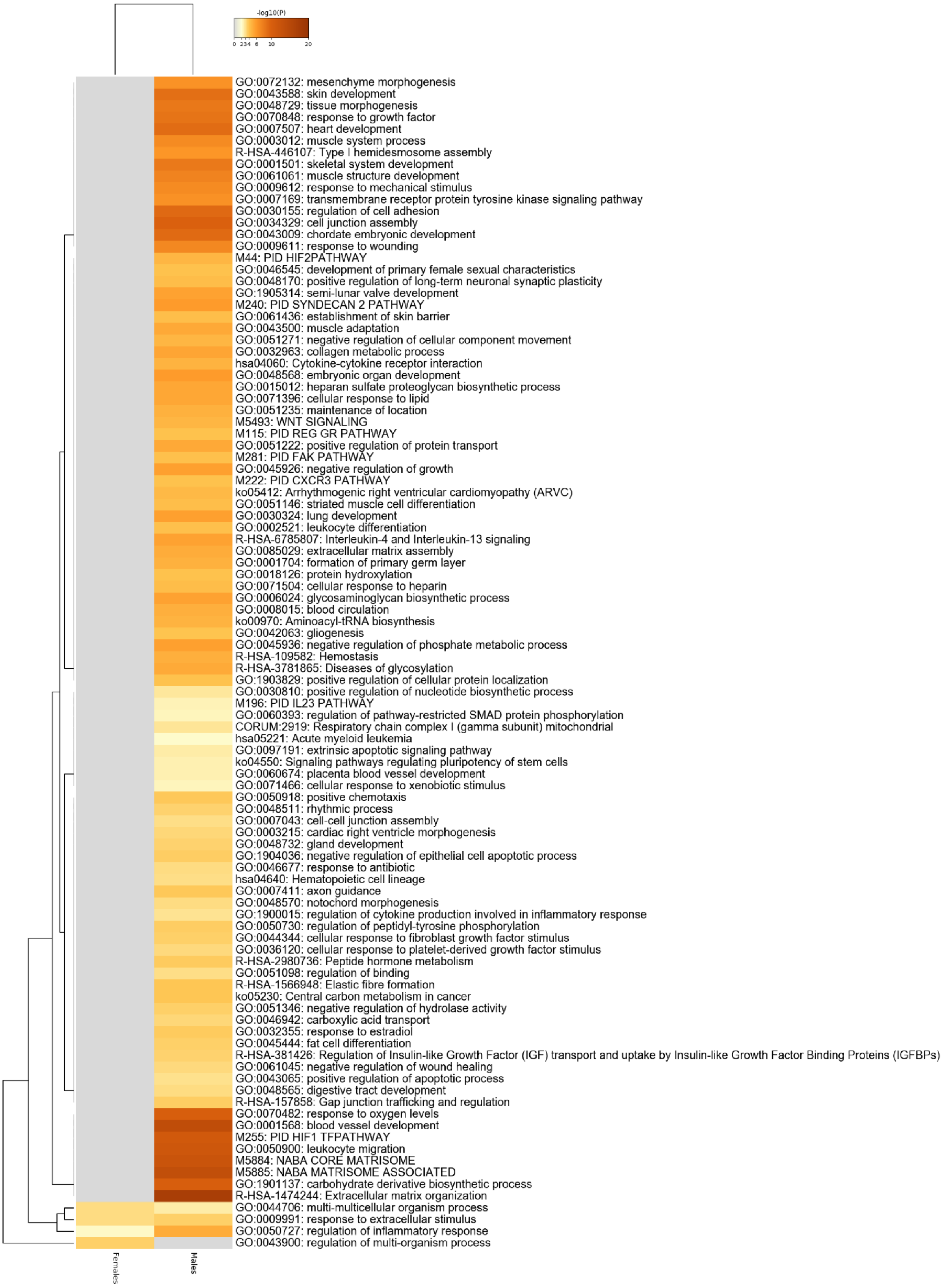

Supplement: Supplementary file 1 — cn3c00621_si_001.pdf [file cn3c00621_si_001.pdf]
